# Revisiting the phylogeny of *Wolbachia* in Collembola

**DOI:** 10.1002/ece3.2738

**Published:** 2017-02-10

**Authors:** Yao Ma, Wan‐Jun Chen, Zhao‐Hui Li, Feng Zhang, Yan Gao, Yun‐Xia Luan

**Affiliations:** ^1^ Key Laboratory of Insect Developmental and Evolutionary BiologyInstitute of Plant Physiology and EcologyShanghai Institutes for Biological SciencesChinese Academy of SciencesShanghaiChina; ^2^ University of Chinese Academy of SciencesBeijingChina; ^3^ Institute of Applied EcologyNanjing Xiaozhuang UniversityNanjingChina; ^4^ Department of EntomologyCollege of Plant ProtectionNanjing Agriculture UniversityNanjingChina

**Keywords:** Collembola, genetic divergence, monophyletic origin, multilocus sequence typing, supergroup E, *Wolbachia*

## Abstract

The endosymbiont *Wolbachia* has been detected in a few parthenogenetic collembolans sampled in Europe and America, including three species of Poduromorpha, two species of Entomobryomorpha, and two species of Neelipleona. Based on 16S rRNA and *ftsZ* gene sequences, most of the *Wolbachia* infecting parthenogenetic collembolans were characterized as members of supergroup E and showed concordant phylogeny with their hosts. However, the two neelipleonan symbionts form another unique group, indicating that *Wolbachia* has infected parthenogenetic collembolans multiple times. In this study, five parthenogenetic collembolan species were identified as hosts of *Wolbachia*, and four new *Wolbachia* strains were reported for four collembolan species sampled in China, respectively, including a neelipleonan strain from *Megalothorax incertus* (*w*Minc). Our results demonstrated that the *Wolbachia* multilocus sequence typing (MLST) system is superior to the 16S rRNA + *ftsZ* approach for phylogenetic analyses of collembolan *Wolbachia*. The MLST system assigned these *Wolbachia* of parthenogenetic collembolans to supergroup E as a unique clade, which included *w*Minc, supporting the monophyletic origin of *Wolbachia* in parthenogenetic collembolan species. Moreover, our data suggested supergroup E as one of the most divergent lineages in *Wolbachia* and revealed the discrepancy between the phylogenies of *Wolbachia* from parthenogenetic collembolans and their hosts, which may result from the high level of genetic divergence between collembolan *Wolbachia*, in association with the geographic differentiation of their hosts, or the possible horizontal transmission of *Wolbachia* between different collembolan species.

## 1 Introduction

*Wolbachia* is an obligate endosymbiont that is widespread in arthropods and filarial nematodes. Prominent effects of *Wolbachia* infection in arthropods include reproductive manipulations, such as feminization of genetic males, parthenogenetic induction, killing of male progeny from infected females, and cytoplasmic incompatibility (Werren, Baldo, & Clark, 2008). Generally, these phenotypes result in an increased frequency of infected females in host populations and thus promote the maternal transmission of *Wolbachia* through generations (LePage & Bordenstein, 2013).

*Wolbachia* cannot be cultivated in vitro; thus, traditional classification methods are ineffective for characterizing *Wolbachia* species. O'Neill, Giordano, Colbert, Karr, and Robertson (1992) proposed the earliest molecular detection method for *Wolbachia* based on the 16S rRNA gene. Molecular typing methods based on more variable genes, such as *ftsZ* (Werren, Zhang, & Guo, 1995) and *wsp* (Zhou, Rousset, & O'Neil, 1998), were developed subsequently. As research progressed, multiple gene sequences were employed to better identify potential supergroups of *Wolbachia*; these analyses usually involved 16S rRNA together with protein‐coding genes such as *dnaA*,* gltA*,* groEL*, and *ftsZ* (Bordenstein & Rosengaus, 2005). However, significant recombination has been reported within *gltA* and *groEL*, as well as between *groEL*‐*ftsZ*,* gltA*‐*dnaA*, and *dnaA*‐*groEL* (Baldo, Bordenstein, Wernegreen, & Werren, 2006). A universal tool for the full characterization of *Wolbachia* using protein‐coding genes has since been developed, that is, the multilocus sequence typing (MLST) scheme for *Wolbachia* (Baldo & Werren, 2007; Baldo et al., 2006). The system involves five housekeeping genes (*gatB*,* coxA*,* hcpA*,* ftsZ*, and *fbpA*) that are broadly distributed across the *w*Mel (*Wolbachia* endosymbiont of *Drosophila melanogaster*) genome as single copies and have been subjected to purifying selection (Baldo et al., 2006). Information on MLST profiles and *Wolbachia* isolates is rapidly growing, and this information is easily accessible in the *Wolbachia* PubMLST database (http://pubmlst.org/wolbachia/). Robust characterization of supergroups is necessary to understand the origin and radiation of *Wolbachia* and to elucidate the nature of the interaction between *Wolbachia* and its hosts (Ros, Fleming, Feil, & Breeuwer, 2009). Thus far, 17 supergroups have been reported for *Wolbachia*, designated A–Q (Augustinos et al., 2011; Bandi, Anderson, Genchi, & Blaxter, 1998; Bing et al., 2014; Bordenstein & Rosengaus, 2005; Glowska, Dragun‐Damian, Dabert, & Gerth, 2015; Haegeman et al., 2009; Lefoulon et al., 2016; Lo, Casiraghi, Salati, Bazzocchi, & Bandi, 2002; Ros et al., 2009; Rowley, Raven, & McGraw, 2004; Vandekerckhove et al., 1999; Werren et al., 1995). However, the proposed supergroup G, based on *wsp* genes only, has been proved to be a *wsp* recombinant clade, instead of a valid supergroup (Baldo & Werren, 2007). Most of the established supergroups infect arthropods, except for supergroups C, D, J, and L. Specifically, supergroups C, D, and J are symbionts of filarial nematodes (Koutsovoulos, Makepeace, Tanya, & Blaxter, 2014), while the sole representative of supergroup L is found in the plant parasitic nematode *Radopholous similis* (Augustinos et al., 2011; Haegeman et al., 2009). Supergroup F has been the only clade found in both filarial nematodes and arthropods to date (Koutsovoulos et al., 2014), while supergroup E is only present in parthenogenetic collembolans (Tanganelli, Fanciulli, Nardi, & Frati, 2014).

As a group of basal hexapods, Collembola (springtails) have been on earth for more than 400 million years. They are small but are abundant in most terrestrial ecosystems, living primarily in the soil and feeding on fungi or decaying plant material (Hopkin, 1997). Collembola includes four orders: Poduromorpha, Entomobryomorpha, Symphypleona, and Neelipleona (Deharveng, 2004). Many collembolan species are considered to be parthenogenetic, as either no males have been sampled in the field (Chahartaghi, Scheu, & Ruess, 2006; Chernova, Potapov, Savenkova, & Bokova, 2009), or reproduction from unfertilized females has been observed in the laboratory (Goto, 1960; Petersen, 1971; Pomorski, 1989). Even in bisexual populations, the sex ratio is often highly biased toward females (Chahartaghi et al., 2006). To date, infection with *Wolbachia* has been reported mainly in parthenogenetic collembolan species, covering Tullbergiidae of Poduromorpha (*Mesaphorura italic*,* Mesaphorura macrochaeta*, and *Paratullbergia callipygos*), Isotomidae of Entomobryomorpha (*Folsomia candida* and *Parisotoma notabilis*), and Neelidae of Neelipleona (*Megalothorax minimus* and *Neelus murinus*) (Czarnetzki & Tebbe, 2004; Tanganelli et al., 2014; Timmermans, Mariën, Roelofs, van Straalen, & Ellers, 2004; Vandekerckhove et al., 1999). A bisexual species, *Orchesella cincta* (Entomobryomorpha, Entomobryidae), also displays *Wolbachia* infection, but the prevalence was very low (Timmermans et al., 2004). In addition, most of the identified host species were collected in Europe (i.e., Germany and Italy), except for an American population of *F. candida*.

Based on a molecular typing system involving 16S rRNA and *ftsZ* genes, collembolan *Wolbachia* fall into three groups: (1) symbionts infecting parthenogenetic springtails of Poduromorpha and Entomobryomorpha, which group together to form supergroup E, showing concordant phylogenetic relationship with their hosts; (2) the strain of bisexual species *O. cincta*, which clusters in supergroup B; and (3) symbionts of the parthenogenetic neelipleonan *Meg. minimus* and *N. murinus*, which form another group (neelid group) occupying a position independent of the D + F + C clade and the H + E + A + B clade (Tanganelli et al., 2014). These findings suggest that there have been multiple occurrences of *Wolbachia* infection in parthenogenetic springtails. However, the neelid group was placed in different positions in trees based on 16S rRNA, *ftsZ*, or 16S rRNA + *ftsZ*; Thus, Tanganelli et al. (2014) defined the neelids as a “group” rather than a “supergroup” and proposed that further studies are necessary to confirm the accuracy of this designation. Currently, there is no MLST information on collembolan *Wolbachia*. With limited data, the phylogenetic position and transmission pattern of collembolan *Wolbachia* are hardly resolved.

In this study, we conducted the first diagnostic screening for *Wolbachia* in collembolan species sampled in China, covering the four orders of Collembola. As a result, we recovered four new *Wolbachia* strains from four parthenogenetic species, including the first case of *Wolbachia* infection in the family Onychiuridae. The phylogenetic positions of these symbionts, as well as the *Wolbachia* from a Danish population of *F. candida* (i.e., *F. candida* DK), were revealed using the *Wolbachia* MLST system and *wsp* gene. Our analyses supported all the newly identified strains belonging to supergroup E, including the one from neelipleonan species, and suggested that the phylogeny of collembolan *Wolbachia* is discordant with that of the hosts. In addition, by analyzing all the known *Wolbachia* of parthenogenetic collembolans, our study revealed the high genetic divergence within supergroup E.

## 2 Materials and methods

### 2.1 Collembolan species

Twelve lines of eleven collembolan species (Table 1) that covers the four orders of Collembola were screened for *Wolbachia* infection. Most species were raised in the laboratory for several generations, except for *Folsomides parvulus* (*Fd. parvulus*) that were collected from a sandy beach on Shengsi Island (Zhejiang, China) and preserved in ethanol. For *F. candida*, two breeding stocks (the DK line from Amsterdam, the Netherlands, and the SH line from Shanghai, China) were tested. Among the 12 screened collembolan lines, *Folsomia candida* (DK), *Mesaphorura yosii*,* Thalassaphorura houtanensis*, and *Megalothorax incertus* were confirmed to be parthenogenetic, through direct observation of reproduction from single unfertilized females in the laboratory, while *Fd. parvulus* and *Arrhopalites minor* are putatively parthenogenetic, as only female specimens were sampled in the field.

**Table 1:** Characterization of collembolan species used in this study

Order	Family	Genus	Species^a^	Parthenogenesis	Source	Geographical origin
Poduromorpha	Hypogastruridae	*Ceratophysella*	*C. denticulata*	No	Laboratory strain	Suzhou, China
	Onychiuridae	*Orthonychiurus*	*O. cf. himalayensis*	No	Laboratory strain	Suzhou, China
		*Thalassaphorura*	***T. houtanensis***	Yes	Laboratory strain	Shanghai, China
	Tullbergiidae	*Mesaphorura*	***Mes. yosii***	Yes	Laboratory strain	Shengsi Island, China
Entomobryomorpha	Isotomidae	*Folsomia*	*F. candida* (SH)	No	Laboratory strain	Shanghai, China
		*Folsomia*	***F. candida*** **(DK)**	Yes	Laboratory strain	Denmark
		*Folsomides*	***Fd. parvulus***	Putative	Field sample	Shengsi Island, China
	Entomobryidae	*Entomobrya*	*E. proxima*	No	Laboratory strain	Shanghai, China
		*Sinella*	*S. curviseta*	No	Laboratory strain	Shanghai, China
		*Lepidocyrtus*	*L. cyaneus*	No	Laboratory strain	Shanghai, China
Neelipleona	Neelidae	*Megalothorax*	***Meg. incertus***	Yes	Laboratory strain	Shanghai, China
Symphypleona	Arrhopalitidae	*Arrhopalites*	*A. minor*	Putative	Laboratory strain	Shanghai, China

a Species infected with *Wolbachia* are indicated in bold.

### 2.2 Diagnostic screening for *Wolbachia* infection in Collembola

The presence of *Wolbachia* was tested via PCR amplification, specifically by targeting the *Wolbachia* 16S rRNA gene. Primary screening was performed on DNA samples extracted from multiple specimens (~100 individuals), with two sets of templates prepared for each collembolan line. For each confirmed host species of *Wolbachia*, the prevalence was calculated according to the screening results from 10 individuals. All extractions were conducted with the Wizard^®^ SV Genomic DNA Purification System (Promega). The primers employed for screenings are listed in Table S1.

### 2.3 Amplification and sequencing of *Wolbachia* and collembolan genes

For each collembolan species carrying *Wolbachia*, we amplified the following genes from single specimens: seven *Wolbachia* genes, including the 16S rRNA gene, five MLST loci (*gatB*,* coxA*,* hcpA*,* ftsZ*, and *fbpA*), and the *wsp* gene; three host genes, including the mt*COI*, 18S, and 28S rRNA genes. The primers employed for each gene are listed in Table S1. The PCR program was set to the following specifications: 3 min at 94°C, followed by 40 amplification cycles (30 s at 94°C, 30 s at the annealing temperature specific to the different primers, and 1 min per thousand base pairs at 72°C) and a 10 min final extension at 72°C.

Most of the PCR products were sequenced directly using the same primers used for amplification, except that some *wsp* genes were cloned for sequencing. Briefly, purified fragments were ligated into the pMD 19‐T vector (Takara, Dalian) and transformed into competent *Escherichia coli* Top10 cells (TIANGEN). Putative positive clones were sequenced with M13 primers (Shanghai Sangon Biotech; Shanghai Sunny Biotechnology). DNA sequences were assembled with the embedded program Seqman from the DNASTAR package (Burland, 2000) and checked with BLAST at NCBI (http://blast.ncbi.nlm.nih.gov/Blast.cgi). Every gene sequence was verified from at least three individuals.

### 2.4 Data assembly and phylogenetic analyses

The phylogenetic status of newly identified *Wolbachia* strains and their cophylogenetic patterns with hosts were examined using four sets of genes: (1) the combined dataset of *Wolbachia* 16S rRNA and *ftsZ* genes, (2) the *Wolbachia* MLST dataset, (3) the *Wolbachia wsp* gene, and (4) the dataset constructed from three host gene sequences (mt*COI*, 18S rRNA, and 28S rRNA). Sequences in addition to our own data were obtained from the *Wolbachia* PubMLST database (data released up to June 2014; no new data available up to June 2016, except for arthropod symbionts of supergroups A, B, and F) and GenBank.

For the dataset of *Wolbachia* 16S rRNA and *ftsZ* genes (Table S2), we chose 46 strains for which both gene sequences were available referring to Tanganelli et al. (2014). Sequences of other nine strains from supergroups A, B, F, I, K, and L were sampled from GenBank to optimize taxon sampling among *Wolbachia* supergroups and host taxonomy. Together with our new data on five *Wolbachia* strains, a total of 60 strains were included in phylogenetic analyses. The dataset of 57 taxa was also analyzed by excluding strains of supergroups I, K, and L, which were not covered in Tanganelli et al. (2014).

For the *Wolbachia* MLST loci and the *wsp* gene, most of the sequences were downloaded from the *Wolbachia* PubMLST database, and *Wolbachia* strains were selected according to their biological information and allelic profiles that are stored in the Isolates database, where each strain is identified by a specific ID number. We first excluded the strains (isolates) for which the supergroup or host genus was not determined. Next, strains with missing MLST loci or *wsp* gene sequences were removed, except for members of supergroups C (isolate 505) and H (isolate 207). For strains showing identical allelic profiles, we conducted additional filtrations based on information on their collection. For example, published strains recovered from the gonads of single infected female specimens derived from lab‐bred species should be considered with priority. Only the strains with the smallest ID numbers were retained if all other information was the same. Additional sequences representing supergroups C (host: *Onchocera ochengi*,* Dirofilaria immitis*) and D (host: *Litomosoides sigmodontis*) were obtained from GenBank and added to the MLST and *wsp* datasets (Table S3).

For the dataset of host genes, the corresponding sequences from one proturan (*Baculentulus tianmushanensis*) and two diplurans (*Lepidocampa weberi* and *Octostigma sinensis*) were retrieved from GenBank and used as outgroups (Table S4).

The nucleotide sequences of each gene were aligned separately on the GUIDANCE web server (Penn et al., 2010) using the algorithm MAFFT (Katoh & Standley, 2013). In the alignment of the *wsp* gene, columns with GUIDANCE scores lower than 0.93 were considered to be unreliable and were removed. For other genes, reliable regions of alignments were selected in GBLOCKS 0.91b (Castresana, 2000), with half of the gap positions being allowed. Finally, the alignments were checked for signs of intragenic recombination using the software package RDP3 (Martin et al., 2010), and recombinants were removed from the MLST and *wsp* datasets (Table S5). The alignments for each multiple‐gene dataset were then concatenated, respectively, using the software BioEdit (Hall, 1999), with missing data assigned with gaps.

PartitionFinder 1.1.1 (Lanfear, Calcott, Ho, & Guindon, 2012) was used to select the best‐fit partitioning schemes and nucleotide substitution models for concatenated datasets and individual protein‐coding genes (Table S6), while the HKY + I + G model was selected for 16S rRNA by jModelTest 2 (Darriba, Taboada, Doallo, & Posada, 2012) on CIPRES (Miller, Pfeiffer, & Schwartz, 2010). Maximum likelihood (ML) analyses were conducted with RAxML 8 (Stamatakis, 2014) on CIPRES, under the recommended GTRGAMMA model, with 1,000 bootstrap replicates. Bayesian analyses were executed in MrBayes 3.2 (Ronquist et al., 2012) with the selected models (Table S6). Each Bayesian analysis was run for 100,000,000 generations, consisting of two parallel runs with four chains each. The best tree was summarized from the last 75% of sampled trees. Resulting trees were visualized with FigTree 1.3.1 (Rambaut, 2009) or iTOL v3 (Letunic & Bork, 2016) and edited in Adobe Illustrator CS6 (Adobe Systems Incorporated).

### 2.5 Estimation of evolutionary divergence for collembolan *Wolbachia*

16S rRNA gene sequence is the only available data for all the seven *Wolbachia* strains reported from parthenogenetic collembolan (Czarnetzki & Tebbe, 2004; Tanganelli et al., 2014; Vandekerckhove et al., 1999). Pairwise genetic distances were estimated for the seven 16S rDNA sequences together with our newly recovered sequences from *Wolbachia* infecting parthenogenetic collembolans in MEGA6 (Tamura, Stecher, Peterson, Filipski, & Kumar, 2013), using the Kimura two‐parameter model with gamma‐shaped rate variation across sites. The input alignment (1,237 nt in length) was generated with the method described above.

In order to further evaluate the divergence among *Wolbachia* of parthenogenetic collembolans, the mean pairwise distance within each *Wolbachia* supergroup was also calculated and compared, based on the alignments of 16S rRNA, *ftsZ* genes, and the MLST system used in phylogenetic analyses, respectively.

## 3 Results

### 3.1 Newly identified collembolan *Wolbachia* strains

Through diagnostic amplification of the *Wolbachia* 16S rRNA gene, we detected *Wolbachia* infection from five parthenogenetic collembolan species of the 11 screened species (12 lines), including one neelipleonan species (Table 1). Each host species displayed a prevalence of 100%. *Wolbachia* infection has been reported previously in *F. candida* (Vandekerckhove et al., 1999); thus, four new host species have been identified in this study. Interestingly, the same sequence was always recovered from different individuals of the same collembolan species, indicating that each species has been infected with a single *Wolbachia* strain. These strains were named after their hosts as follows: *w*Fcan, *w*Myos, *w*Minc, *w*Fpar, and *w*Thou infected *F. candida* (DK), *Mes. yosii*,* Meg. incertus*,* Fd. parvulus*, and *T. houtanensis*, respectively.

Nearly full‐length 16S rRNA gene sequences (approximately 1,400 bp) and partial sequences of the five *Wolbachia* MLST genes (*gatB*, 412–963 bp; *coxA*, 461–530 bp; *hcpA*, 495–557 bp; *ftsZ*, 912–962 bp; and *fbpA*, 486–700 bp) were obtained for all five strains, while *wsp* gene sequences were acquired only for *w*Fcan (723 bp), *w*Myos (633 bp), and *w*Minc (519 bp). For each *Wolbachia* gene, the sequences were different among strains from different species, suggesting that each collembolan species was infected with a unique *Wolbachia* strain.

All of these sequences were submitted to GenBank (Accession Nos. KT799584‐KT799616), and the sequences of the MLST loci were accepted by the PubMLST database as well (Table S3).

### 3.2 Phylogenetic analyses of collembolan *Wolbachia*

The alignment of the data assembly was 1,946 nt for the 60‐taxon dataset of 16S rRNA and *ftsZ* genes (1,241 nt for 16S rRNA, 705 nt for *ftsZ*); 1,856 nt for the 57‐taxon dataset of 16S rRNA + *ftsZ* (1,187 nt for 16S rRNA, 669 nt for *ftsZ*) (Table S2); 2,076 nt with 145 taxa for the MLST scheme (402, 429, 435, 369, and 441 sites for *coxA*,* fbpA*,* ftsZ*,* gatB*, and *hcpA*, respectively); and 453 nt with 117 taxa for the *wsp* gene.

No matter if supergroups I, K, and L were included or not, the phylogenies inferred from the concatenated datasets of 16S rRNA and *ftsZ* genes including all 11 *Wolbachia* strains of parthenogenetic collembolans were consistent with the results of Tanganelli et al. (2014): An independent clade was recovered for the three neelipleonan *Wolbachia* strains, including *w*Minc, which was apart from supergroup E, the clade for other symbionts of parthenogenetic collembolans (Fig. S7A,B). *w*Minc itself could also form an independent clade, when six previously reported *Wolbachia* strains of parthenogenetic collembolans were excluded from analyses (Fig. S7C). Moreover, in the gene tree with 60 taxa, the “neelid group” was grouped with supergroups K and L (Fig. S7A). However, single‐gene trees based on 16S rRNA and *ftsZ* showed different positions for collembolan *Wolbachia* strains with low support values (Fig. S7D,E).

**Figure 1:**
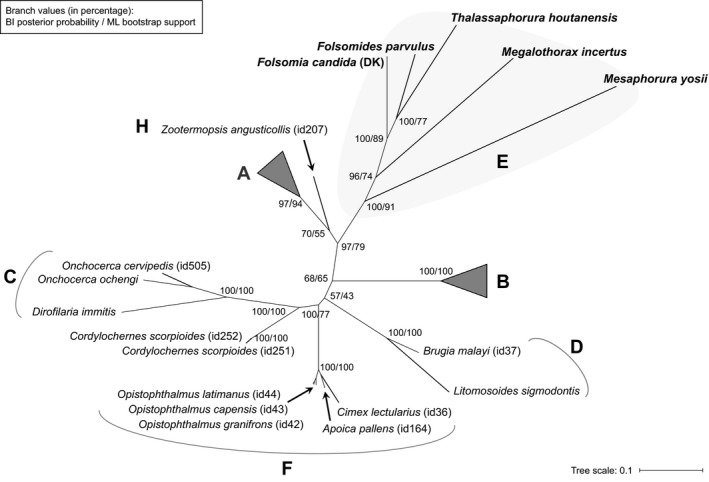
Unrooted *Wolbachia* tree based on the concatenated dataset of five MLST loci. The dendrogram was generated through Bayesian inference (BI). The same tree topology was recovered in the maximum likelihood (ML) analysis. Support values at nodes indicate Bayesian posterior probabilities (left) and ML bootstraps (right) as percentages. A partitioning scheme based on codon positions across genes was used in both inferences. *Wolbachia* strains are represented with the name of their host species, followed by their isolate ID in the PubMLST database (in parentheses). Collembolan symbionts are shown in bold and enlarged fonts. The corresponding clade for supergroup E is marked with gray shading. Supergroups A and B are collapsed to simplify the display of the tree. There are 82 (isolate ID: 1–18, 38, 46, 55, 61, 68, 78, 88, 96, 98, 103, 104, 106, 107, 108, 110–114, 116, 117, 120–122, 126, 127, 129, 133, 135, 137–145, 165, 167–171, 177, 179, 182, 183, 250, 294, 325, 346–352, 399, 401, 420, 425, 555, 613) and 45 (isolate ID: 19, 20, 21, 25–27, 29, 31–33, 35, 40, 70, 87, 97, 102, 118, 130, 132, 194, 195, 200, 208, 212, 219, 225, 235, 246, 267–270, 293, 309, 310, 315, 317, 318, 353, 454, 456, 457, 468, 507, 1595) taxa in clades A and B, respectively

ML and Bayesian analyses of the *Wolbachia* MLST system resulted in a new robust phylogeny: All of the collembolan *Wolbachia* strains identified in this study were grouped into a unique lineage as supergroup E, including *w*Minc, with high support values (ML: 91, Bayesian: 100; in percentage). All supergroups were distinguished from each other, and supergroup E was the sister‐group to clade A + H (Figure 1). This monophyletic clade for collembolan *Wolbachia* was consistently recovered in phylogenies of individual genes, although topologies of these trees showed some differences (Fig. S8).

The *wsp* phylogeny further confirmed that the clade recovered from parthenogenetic collembolan species was monophyletic, by placing *w*Minc in supergroup E (Fig. S9). In addition, none of the collembolan *Wolbachia* sequences were identified as an outcome of recombination between any other sequences in the MLST and *wsp* datasets (Table S5).

In conclusion, inferences based on 16S rRNA and *ftsZ* genes were inadequate for full characterization of neelipleonan *Wolbachia*. With the MLST system, this group was assigned to supergroup E together with other parthenogenetic collembolan *Wolbachia*.

### 3.3 Phylogenetic comparison between *Wolbachia* and Collembola

**Figure 2:**
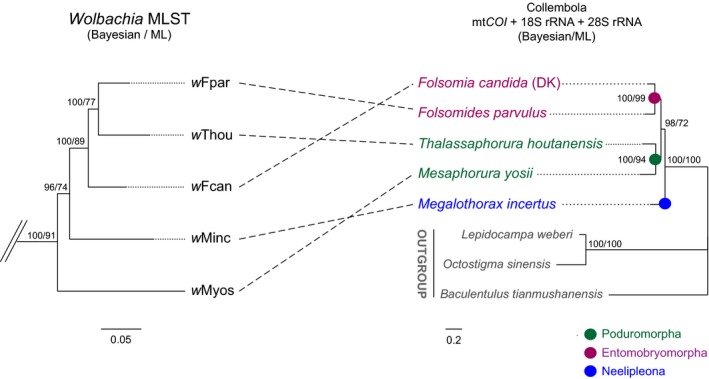
Comparison between the phylogenies of the *Wolbachia* stains recovered in this study and their parthenogenetic collembolan hosts. The phylogeny of collembolan *Wolbachia* (left) was derived from the MLST tree (Figure 1). The phylogenetic relationships among host species (right) were examined with three outgroups (the proturan species *Baculentulus tianmushanensis* and two dipluran species, *Lepidocampa weberi* and *Octostigma sinensis*). The same tree topology was obtained from Bayesian and ML inferences based on the concatenated dataset of mt*COI*, 18S rRNA, and 28S rRNA gene sequences. Collembolans are color‐coded taxonomically at the level of orders

Although supergroup E was distinct and monophyletic, its members did not cluster with their hosts taxonomically. In the concatenated MLST phylogeny (Figure 2), *w*Thou infecting *T. houtanensis* (Poduromorpha) was the sister‐group of *w*Fpar infecting *Fd. parvulus* (Entomobryomorpha), instead of *w*Fcan infecting *F. candida* (DK) (Entomobryomorpha). Moreover, *w*Myos infecting *Mes. yosii* (Poduromorpha) was the sister‐group to all other strains in clade E. This divergence was confirmed by phylogenetic analysis of the mt*COI*, 18S rRNA, and 28S rRNA genes of the hosts, using proturan and dipluran species as outgroups (Figure 2). The combined dataset of these genes (2,730 nt and 8 taxa, including 654 nt for mt*COI*, 1,661 nt for 18S rRNA, and 415 nt for 28S rRNA) clustered perfectly for the five host species according to their taxonomic status at the order level. In other words, species from the orders Poduromorpha (*F. candida* (DK) and *Fd. parvulus*) and Entomobryomorpha (*Mes. yosii* and *T. houtanensis*) were grouped separately in the tree (Figure 2).

### 3.4 Genetic divergence of *Wolbachia* from parthenogenetic collembolans

**Figure 3:**
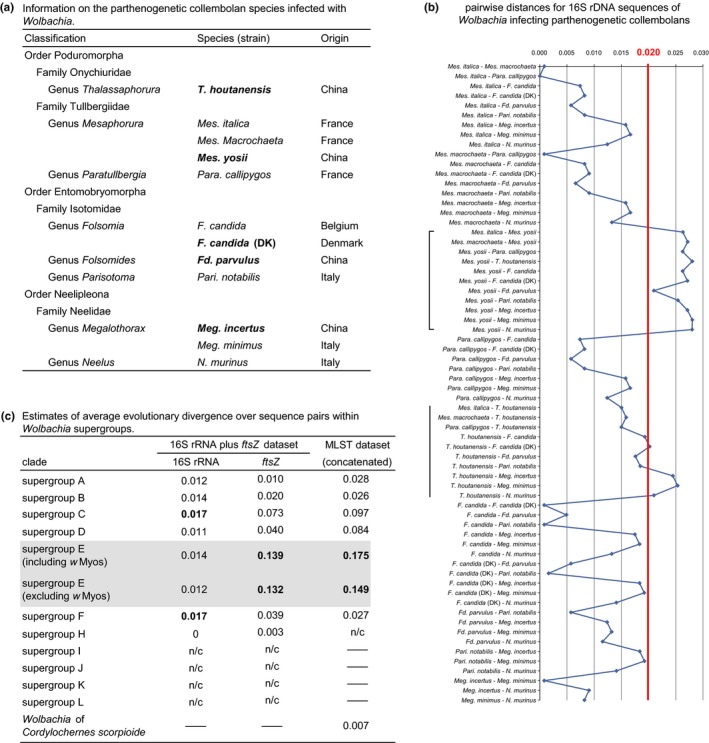
Genetic divergence between the *Wolbachia* infecting different parthenogenetic collembolan species. (a) Information on all the known parthenogenetic collembolans infected with *Wolbachia*. Host species (stocks) identified in this study are indicated in bold. *Wolbachia* of *Mes. Macrochaeta* was not included in our phylogenetic analyses (referring to Fig. S7), because its *ftsZ* gene sequence is not available. The GenBank accession number for its 16S rDNA sequence is AJ422184. (b) Pairwise distances for the 16S rRNA gene sequences of 12 *Wolbachia* strains infecting parthenogenetic collembolans are shown in the line graph. *Wolbachia* strains are represented with the name of their host species. The scale value of 0.02 is marked with a red line, because the value was considered a threshold to establish new supergroups for *Wolbachia* (Augustinos et al., 2011). Sequence pairs concerning about *w*Myos infecting *Mes. yosii* and *w*Thou infecting *T. houtanensis* are indicated with big square bracket and vertical line, respectively. (c) The average evolutionary divergence over sequence pairs within each *Wolbachia* supergroups was calculated, using 16S rRNA,* ftsZ*, and MLST scheme, respectively. The values of distances are shown with three digits after the decimal point, and the maximums are indicated in bold. The “n/c” denotes that the value cannot be calculated due to only one related sequence available in the supergroup. The “—” indicates the absence of the related sequence in the supergroup

Among all the known *Wolbachia* of parthenogenetic collembolans (Figure 3a), the 16S rRNA gene sequences of *w*Myos infecting *Mes. yosii* and *w*Thou infecting *T. houtanensis* are highly divergent (Figure 3b). Particularly, *w*Myos displays a distance exceeding 2% compared with any other symbionts of parthenogenetic collembolans (Figure 3b). On the contrast, genetic distances of the *Wolbachia* recovered from three neelipleonan species (*Meg. minimus*,* Meg. incertus* and *Neelus murinus*) are <2% from most of the other symbionts, except for comparisons with *w*Myos and *w*Thou (Figure 3b). The genetic distance of 16S rRNA sequences >2% was considered necessary for establishing the new supergroup for *Wolbachia* (Augustinos et al., 2011); thus, the previously proposed “neelid group” was not supported.

In addition, compared with other *Wolbachia* supergroups, the average genetic distance of 16S rDNA within supergroup E is moderately divergent; however, its average genetic distances of protein‐coding genes are much higher, no matter whether *w*Myos is included (Figure 3c), suggesting that supergroup E is the most divergent lineage in *Wolbachia*.

## 4 Discussion

In this study, all the *Wolbachia* recovered from parthenogenetic collembolans, including a strain from neelipleonan species, were assigned to supergroup E with the five MLST loci (Figure 1). On the contrast, the typing system based on 16S rRNA and *ftsZ* genes is insufficient in identifying supergroup E members for the following reasons. First, the 16S rRNA gene is highly conserved, with an average diversity of 3.61% between supergroups (Ros et al., 2009). The missing first 400 sites of the two available sequences from supergroup H in the 16S rRNA alignment further reduced the divergence between members of supergroups H and E, leading to a mixed clade of these groups in the phylogenetic tree of 16S rRNA (Fig. S7D). Second, the *ftsZ* sequences for the two reported strains of neelid group (*Wolbachia* of *N. murinus* and *Meg. minimus*) are only 480 bp in length and provide only 5/7 sites in the *ftsZ* alignment, while other E‐clade members, as well as the related lineages such as supergroup H, K, and L, have few missing data. Third, the 16S rRNA and *ftsZ* phylogeny suggest inconsistent positions for neelipleonan *Wolbachia* strains (Fig. S7D,E).

With new data of collembolan *Wolbachia* that were recovered from Chinese springtails, we observed discordance between the phylogenies of supergroup E members and their hosts (Figure 2). A main cause of the non‐matching phylogenies should be the position of *w*Myos, which is the sister‐group to all other strains in clade E in MLST tree (Figure 1). As *w*Myos is the most divergent strain in supergroup E (Figure 3), its position in trees may be affected by long‐branch attraction, and its variation is probably related to the special habitat of its host (a sandy beach on an island). Generally, discordant phylogenies of hosts and *Wolbachia* are explained by horizontal transmission of *Wolbachia* (Ferri et al., 2011; Werren et al., 1995), and it might be the case in collembolan *Wolbachia* (Timmermans et al., 2004). However, in this study, no intragenic recombination of MLST and *wsp* genes was detected within supergroup E, as well as between supergroup E and other supergroups, so we have not found solid evidence to support the hypothesis on horizontal transmission of *Wolbachia* between different collembolan species.

Through PCR screening for *Wolbachia* in collembolan species sampled in China, we further confirmed that *Wolbachia* tend to live symbiotically with parthenogenetic collembolans. Among the 11 tested Chinese populations, only four parthenogenetic ones are infected with *Wolbachia*. However, the putative parthenogenetic species *A. minor* (Symphypleona: Arrhopalitidae) is free of *Wolbachia* (Table 1). The absence of *Wolbachia* infection in parthenogenetic collembolans has been evidenced in several species of Poduromorpha, Entomobryomorpha, and Symphypleona (Tanganelli et al., 2014). Whether it is a secondary loss still needs further study covering more data on collembolan *Wolbachia*.

Most of the identified collembolan *Wolbachia* endosymbionts have been recovered from parthenogenetic species and belong to supergroup E, except for a B‐type strain that infects *O. cincta*. Considering the extremely low prevalence reported in *O. cincta* (Timmermans et al., 2004), retracing the origin of the symbiont is a difficult task. In our unrooted MLST tree, supergroup E is sister to the A + H clade; however, the sister‐group relationship between A and H is not well supported (Figure 1). As supergroup H was considered the sister‐group of supergroup E in most of the previous studies (Bordenstein et al., 2009; Lefoulon et al., 2016; Lo et al., 2007), the relationship between A, H, and E would be better illustrated when more MLST sequences are available, especially for supergroup H. The phylogenetic inferences based on 16S rRNA and *ftsZ* genes indicate that supergroups K and L might also be close to supergroup E (Fig. S7A). Based on rooted phylogenies, a recent phylogenomic study of *Wolbachia* supergroup relationships proposed that supergroup E, represented by the endosymbiont of *F. candida*, is the sister‐group to all other studied supergroups within genus *Wolbachia* (Gerth, Gansauge, Weigert, & Bleidorn, 2014). However, the conclusion was drawn out when only one supergroup E strain was analyzed, insufficient data for the coherent lineage supergroup H were used (only one representative, with 19 genes acquired of 90 targeted), and no data for supergroups K and L were involved. In our opinion, the phylogenetic status of supergroup E would be better resolved with genomic data from more collembolan symbionts, as well as additional data from closely related clades, such as supergroups H, K, and L.

In conclusion, we enriched the data of collembolan *Wolbachia* by diagnostic screening in Chinese collembolans for the first time and revisited the supergroup status of *Wolbachia* from parthenogenetic collembolans. Using the *Wolbachia* MLST system, we support the hypothesis of monophyletic origin of symbionts for parthenogenetic collembolan species by designating all five *Wolbachia* strains recovered in this study to supergroup E, including the neelipleonan *Wolbachia w*Minc. The lack of intragenic recombination in MLST and *wsp* genes between supergroup E and other supergroups further supports supergroup E as a unique clade. Particularly, high genetic divergence within supergroup E is identified, suggesting supergroup E as one of the most divergent lineages in *Wolbachia*. In addition, the inconsistency between the phylogenies of *Wolbachia* and parthenogenetic collembolans is newly discovered, which might be a sign of *Wolbachia* horizontal transmission between different hosts, but on the other hand, it might be caused by the highly differentiated collembolan *Wolbachia* strain, *w*Myos, for which host species was collected from a special habitat: a sandy beach on an island.
