# Supplementary material for: Revisiting the phylogeny of Wolbachia in Collembola
Source: Ecol Evol. 2017 Feb 10;7(7):2009–17. doi: 10.1002/ece3.2738 (PMC5383468; doi:10.1002/ece3.2738)
Supplement: Supplementary file 1 [file ECE3-7-2009-s001.docx]

**Table S1.** List of primers used in this study.

| Primer ^a^ | Sequence | pairing with | Reference ^c^ |  |
| --- | --- | --- | --- | --- |
| 16S rRNA (*Wolbachia*) |  |  |  |  |
| 16SW_F ^b^ | 5'-TTGTAGCCTGCTATGGTATAACT-3' | 16SW_R, 16SAr | O'Neill et al. (1992) |  |
| 16SW_R ^b^ | 5'-GAATAGGTATGATTTTCATGT-3' | 16SW_F, Ec16s-45f | O'Neill et al. (1992) |  |
| 16SAr ^b^ | 5'-TAAGGGATTAGCTTAGCCTC-3' | 16SW_F | Werren et al. (1995) |  |
| 16SAf | 5'-TTCGGCCGGGTTTCACACAG-3' | Ec16s-1511r | Werren et al. (1995) |  |
| Ec16s-45f | 5'-GCTTAACACATGCAAG-3' | 16SW_R | Tanganelli et al. (2014) |  |
| Ec16s-1511r | 5'-CCTTGTTACGACTTCACCCCAG-3' | 16SAf |  |  |
| *gatB* (*Wolbachia*) |  |  |  |  |
| gatB_F1 | 5'-GAKTTAAAYCGYGCAGGBGTT-3' | gatB_R1 | *Wolbachia* MLST Database |  |
| gatB_R1 | 5'-TGGYAAYTCRGGYAAAGATGA-3' | gatB_F1 | (http://pubmlst.org/wolbachia/) |  |
| gatB_F2adp | 5'-TGTAAAACGACGGCCAGTCAGATAACNCARTTYTTYGARCC-3' | gatB_R2adp |  |  |
| gatB_R2adp | 5'-CAGGAAACAGCTATGACCATTGTTCCATCNACDATRAARTC-3' | gatB_F2adp |  |  |
| gatB-F-181 | 5'-CAAGCAATAYGYACYGGTCT-3' | gatB-R-1162 | this study |  |
| gatB-R-1162 | 5'-GYTTRCCAAGTTTAGCAGAG-3' | gatB-F-181 |  |  |
| *coxA* (*Wolbachia*) |  |  |  |  |
| coxA_F1 | 5'-TTGGRGCRATYAACTTTATAG-3' | coxA_R1 | *Wolbachia* MLST Database |  |
| coxA_R1 | 5'-CTAAAGACTTTKACRCCAGT-3' | coxA_F1, coxA_AspecF1 |  |  |
| coxA_AspecF1 | 5'-ATACCCACCTTTATCACAGG-3' | coxA_R1 |  |  |
| coxA_F2adp | 5'-TGTAAAACGACGGCCAGTGGAGGATTYGGNAAYTGGTTYGT-3' | coxA_R2adp |  |  |
| coxA_R2adp | 5'-CAGGAAACAGCTATGACCCCACCCCACATNGTNGCDATCCA-3' | coxA_F2adp |  |  |
| *hcpA* (*Wolbachia*) |  |  |  |  |
| hcpA_F1 | 5'-GAAATARCAGTTGCTGCAAA-3' | hcpA_R1 | *Wolbachia* MLST Database |  |
| hcpA_R1 | 5'-GAAAGTYRAGCAAGYTCTG-3' | hcpA_F1 |  |  |
| hcpA_F3 | 5'-ATTAGAGAAATARCAGTTGCTGC-3' | hcpA_R3 |  |  |
| hcpA_R3 | 5'-CATGAAAGACGAGCAARYTCTGG-3' | hcpA_F3 |  |  |
|  |  |  |  |  |
| **Table S1.** Continued. |  |  |  |  |
| Primer ^a^ | Sequence | pairing with | Reference ^c^ |  |
| hcpA-F-32 | 5'-AACATCGGAAAGGYGCTCAG-3' | hcpA_AspecR1 | this study |  |
| hcpA_AspecR1 | 5'-TTCTARYTCTTCAACCAATGC-3' | hcpA-F-32 | *Wolbachia* MLST Database |  |
| *ftsZ* (*Wolbachia*) |  |  |  |  |
| ftsZf1 | 5'-GTTGTCGCAAATACCGATGC-3' | ftsZcol-r | Werren et al. (1995) |  |
| ftsZcol-r | 5'-CCTTCACTTCCCTGCTCAG-3' | ftsZf1 | Czarnetzki and Tebbe (2004) |  |
| ftsZ-TU-F72 | 5'-TGGTGCTTTACCTGATGTTG-3' | ftsZ-TU-R825 | this study |  |
| ftsZ-TU-R825 | 5'-CAGAAACTCTAACTCTTCCCTCC-3' | ftsZ-TU-F72 |  |  |
| ftsZ_AspecF1 | 5'-AAAGATAGTCATATGCTTTTC-3' | ftsZ_R1 | *Wolbachia* MLST Database |  |
| ftsZ_F1 | 5'-ATYATGGARCATATAAARGATAG-3' | ftsZr1 |  |  |
| ftsZ_R1 | 5'-TCRAGYAATGGATTRGATAT-3' | ftsZ_AspecF1 |  |  |
| ftsZr1 | 5'-GTTAAGTAAGCTGGTATATC-3' | ftsZ_F1 | Werren et al. (1995) |  |
| *fbpA* (*Wolbachia*) |  |  |  |  |
| fbpA_F2adp | 5'-TGTAAAACGACGGCCAGTGTAGATCARGGNTTYGARCAYGG-3' | fbpA_R2adp | *Wolbachia* MLST Database |  |
| fbpA_R2adp | 5'-CAGGAAACAGCTATGACCTTACCGCCACCYTGYTTDATYTC-3' | fbpA_F2adp |  |  |
| fbpA_F3 | 5'-GTTAACCCTGATGCYYAYGAYCC-3' | fbpA_R3 |  |  |
| fbpA_R3 | 5'-TCTACTTCCTTYGAYTCDCCRCC-3' | fbpA_F3 |  |  |
| *wsp* (*Wolbachia*) |  |  |  |  |
| 136F-5end-r | 5'-AAYGGTGAACTTTTACCTYT-3' | 691R, wsp-wMel-R590 | this study |  |
| 691R | 5'-AAAAATTAAACGCTACTCCA-3' | 136F-5end-r, wsp-wMel-F247 | Zhou et al. (1998) |  |
| wsp-wMel-F247 | 5'-AAAATGGAYGACATYAGRGTT-3' | 691R | this study |  |
| wsp-wMel-R590 | 5'-CCATAAGAACCRAAATARCGAG-3' | 136F-5end-r |  |  |
| Wsp-F-30mers | 5'-TGGTCCAATAAGTGATGAAGAAACTAGCTA-3' | Wsp-R-30mers | Jeyaprakash et al. (2000) |  |
| Wsp-R-30mers | 5'-AAAAATTAAACGCTACTCCAGCTTCTGCAC-3' | Wsp-F-30mers |  |  |
|  |  |  |  |  |
| **Table S1.** Continued. |  |  |  |  |
| Primer ^a^ | Sequence | pairing with | Reference ^c^ |  |
| mt*COI* (collembolan) |  |  |  |  |
| LCO | 5'-GGTCAACAAATCATAAAGATATTGG-3' | HCO | Folmer et al. (1994) |  |
| HCO | 5'-TAAACTTCAGGGTGACCAAAAAATCA-3' | LCO |  |  |
| 18S rRNA (collembolan) |  |  |  |  |
| 18S1L | 5'-TACCTGGTTGATCCTGCCAGT-3' | 18S1R | Luan et al. (2005) |  |
| 18S1R | 5'-TAATATACGCTATTGGAGCTGG-3' | 18S1L |  |  |
| 18SL500 | 5'-GTTCGATTCCGGAGAGGGAG-3' | 18SR1470 |  |  |
| 18SR1470 | 5'-TTAGAACTAGGGCGGTATCTG-3' | 18SL500 |  |  |
| 18SL1210 | 5'-CCTTGAGAAAATTGGAGTGCT-3' | 18S3R |  |  |
| 18S3R | 5'-CCTACGGAAACCTTGTTACG-3' | 18SL1210 |  |  |
| 28S rRNA (collembolan) |  |  |  |  |
| 28Sf | 5'-TGGGACCCGAAAGATGGTG-3' | 28S5b |  |  |
| 28S5b | | 5'-ACACACTCCTTAGCGGA-3' | 28Sf |  |
| a. Primers with "adp" in their names could be sequenced with M13 sequencing tags. | |  |  |  |
| b. Primers used in screening for *Wolbachia* infection. | |  |  |  |
| c. Full information on the references is listed in Supplementary File 10. | |  |  |  |

**Table S2.** *Wolbachia* strains in the phylogenetic trees based on 16S rRNA and *ftsZ* gene sequences.

| Host | | | |  | GenBank Accession Number ^a^ | | Supergroup |
| --- | --- | --- | --- | --- | --- | --- | --- |
| Phylum | Class | Order | Species |  | 16S rRNA | *ftsZ* |  |
| Arthropoda | Arachnida | Prostigmata | *Bryobia spec.* V |  | EU499316 | EU499321 | K |
|  | Collembola | Entomobryomorpha | *Folsomia candida* |  | AF179630 | AY326459 | E |
|  |  |  | ***Folsomia candida* (DK)** |  | **KT799585** | **KT799600** | E |
|  |  |  | ***Folsomides parvulus*** |  | **KT799586** | **KT799601** | E |
|  |  |  | *Orchesella cincta* |  | (personal communication) | | B |
|  |  |  | *Parisotoma notabilis* |  | KC767947 | KC767950 | E |
|  |  | Neelipleona | ***Megalothorax incertus*** |  | **KT799584** | **KT799599** | E |
|  |  |  | *Megalothorax minimus* |  | KC767945 | KC767948 | Neelid group ^b^ |
|  |  |  | *Neelus murinus* |  | KC767946 | KC767949 | Neelid group ^b^ |
|  |  | Poduromorpha | *Mesaphorura italica* |  | AJ575104 | AJ575103 | E |
|  |  |  | ***Mesaphorura yosii*** |  | **KT799588** | **KT799603** | E |
|  |  |  | *Paratullbergia callipygos* |  | AJ509026 | AJ575101 | E |
|  |  |  | ***Thalassaphorura houtanensis*** |  | **KT799587** | **KT799602** | E |
|  | Insecta | Diptera | *Culex pipiens* |  | X61768 | U28209 | B |
|  |  |  | *Drosophila melanogaster* |  | NC_002978 | | A |
|  |  |  | *Drosophila sechellia* |  | U17059 | U28179 | A |
|  |  |  | *Drosophila simulans* *w*Ha |  | NC_021089 | | A |
|  |  |  | *Drosophila simulans* *w*No |  | NC_021084 | | B |
|  |  |  | *Drosophila simulans* *w*Ri |  | DQ412085 | U28178 | A |
|  |  |  | *Phlebotomus papatasi* |  | U80584 | U80585 | A |
|  |  |  | *Thecodiplosis japonensis* |  | AF220604 | AF220605 | A |
|  |  | Hemiptera | *Cimex lectularius* |  | AY316361 | AY316362 | F |
|  |  |  |  |  |  |  |  |
| **Table S2.** Continued. | | | | | | | |
| Host | | | |  | GenBank Accession Number ^a^ | | Supergroup |
| Phylum | Class | Order | Species |  | 16S rRNA | *ftsZ* |  |
| Arthropoda | Insecta | Hemiptera | *Laodelphax striatellus* |  | AB039036 | AB039038 | B |
|  |  |  | *Oeciacus vicarius* |  | AY091456 | AY091457 | F |
|  |  |  | *Sogatella furcifera* |  | AB039037 | AB039039 | B |
|  |  | Hymenoptera | *Encarsia formosa* |  | AF045189 | U28196 | B |
|  |  |  | *Muscidifurax uniraptor* |  | L02882 | U28186 | A |
|  |  |  | *Nasonia giraulti* |  | M84690 | U28203 | B |
|  |  |  | *Nasonia longicornis* ^c^ |  | M84691 | DQ842331 | A |
|  |  |  | *Nasonia longicornis* ^c^ |  | M84692 | U28204 | B |
|  |  |  | *Nasonia vitripennis* ^c^ |  | M84687 | U28188 | A |
|  |  |  | *Nasonia vitripennis* ^c^ |  | M84686 | U28205 | B |
|  |  |  | *Trichogramma cordubensis* |  | L02883 | U28200 | B |
|  |  |  | *Trichogramma deion* |  | L02884 | U28201 | B |
|  |  | Isoptera | *Coptotermes lacteus* |  | DQ837198 | DQ837189 | F |
|  |  |  | *Kalotermes flavicollis* |  | Y11377 | AJ292345 | F |
|  |  |  | *Microcerotermes* sp. |  | AJ292347 | AJ292346 | F |
|  |  |  | *Serritermes serrifer* |  | DQ837205 | DQ837193 | ? |
|  |  |  | *Zootermopsis angusticollis* |  | AY764279 | AY764283 | H |
|  |  |  | *Zootermopsis nevadensis* |  | AY764280 | AY764284 | H |
|  |  | Lepidoptera | *Ephestia kuehniella* |  | AB360384 | U62125 | A |
|  |  | Orthoptera | *Gryllus integer* |  | U83094 | AF011269 | B |
|  |  |  | *Gryllus pennsylvanicus* |  | U83090 | U28195 | B |
|  |  |  | *Gryllus rubens* |  | U83092 | U83102 | B |
|  |  | Siphonaptera | *Ctenocephalides felis* |  | AY335923 | AJ628415 | I |
|  |  |  |  |  |  |  |  |
| **Table S2.** Continued. | | | | | | | |
| Host | | | |  | GenBank Accession Number ^a^ | | Supergroup |
| Phylum | Class | Order | Species |  | 16S rRNA | *ftsZ* |  |
| Arthropoda | Malacostraca | Isopoda | *Armadillidium vulgare* |  | AJ223238 | AJ223243 | B |
| Nematoda | Chromadorea | Spirurida | *Brugia malayi* |  | AF051145 | AJ010269 | D |
|  |  |  | *Brugia phahangi* |  | AJ012646 | AJ010270 | D |
|  |  |  | *Dipetalonema gracile* |  | AJ548802 | FR827924 | J |
|  |  |  | *Dirofilaria immitis* |  | AF088187 | AJ010272 | C |
|  |  |  | *Dirofilaria repens* |  | AJ276500 | AJ010273 | C |
|  |  |  | *Litomosoides sigmodontis* |  | AF069068 | AJ010271 | D |
|  |  |  | *Mansonella perforata* |  | FR827939 | FR827926 | F |
|  |  |  | *Mansonella* sp. |  | AJ628417 | AJ628414 | F |
|  |  |  | *Onchocerca gibsoni* |  | AJ276499 | AJ010267 | C |
|  |  |  | *Onchocerca gutturosa* |  | AJ276498 | AJ010266 | C |
|  |  |  | *Onchocerca ochengi* |  | AJ010276 | AJ010268 | C |
|  |  |  | *Onchocerca volvolus* |  | AF069069 | AF282845 | C |
|  |  |  | *Wuchereria bancrofti* |  | AF093510 | AF081198 | D |
|  |  | Tylenchida | *Radopholus similis* |  | EU833482 | EU833483 | L |
| a. Sequences obtained in this study were shown in bold, and their supergroups were designated according to the MLST phylogeny. | | | | | | | |
| b. "Neelid group", the putative group for *Wolbachia* endosymbionts of neelipleonan species *Megalothorax minimus* and *Neelus murinus* was proposed by Tanganelli et al. (2014). | | | | | | | |
| c. The *Nasonia* wasps *N. longicornis* and *N. vitripennis* showed double infections with A-type and B-type *Wolbachia* strains (Werren et al. 1995). | | | | | | | |

**Table S3**. *Wolbachia* strains without PubMLST records and those identified in this study using in the phylogenetic trees based on MLST and *wsp* gene sequences. ^a^

| Host | | | | Supergroup | Accession number of sequences ^d^ | | | | | |
| --- | --- | --- | --- | --- | --- | --- | --- | --- | --- | --- |
| Species or subspecies ^b^ | Class | Order | Phenotype ^c^ |  | *coxA* | *fbpA* | *ftsZ* | *gatB* | *hcpA* | *wsp* |
| *Dirofilaria immitis* | Chromadorea | Spirurida | M | C | FJ390244 | —— | AJ010272 | HM768893 | —— | AJ252062 |
| *Onchocerca ochengi* | Chromadorea | Spirurida | M | C | NC_018267 | | | | | |
| *Litomosoides sigmodontis* | Chromadorea | Spirurida | M | D | FJ390171 | AF409112 | JQ888344 | FJ390246 | AJ010271 | AF409112 |
| ***Folsomia candida* DK** | Collembola | Entomobryomorpha | PI | E | KT799590 | KT799595 | KT799600 | KT799605 | KT799610 | KT799615 |
|  |  |  |  |  | (148) | (227) | (135) | (171) | (179) |  |
| ***Folsomides parvulus*** | Collembola | Entomobryomorpha | P | E | KT799591 | KT799596 | KT799601 | KT799606 | KT799611 | —— |
|  |  |  |  |  | (197) | (385) | (174) | (213) | (224) |  |
| ***Thalassaphorura houtanensis*** | Collembola | Poduromorpha | P | E | KT799592 | KT799597 | KT799602 | KT799607 | KT799612 | —— |
|  |  |  |  |  | (233) | (418) | (211) | (251) | (279) |  |
| ***Mesaphorura yosii*** | Collembola | Poduromorpha | P | E | KT799593 | KT799598 | KT799603 | KT799608 | KT799613 | KT799616 |
|  |  |  |  |  | (195) | (383) | (172) | (211) | (225) |  |
| ***Megalothorax incertus*** | Collembola | Neelipleona | P | E | KT799589 | KT799594 | KT799599 | KT799604 | KT799609 | KT799614 |
|  |  |  |  |  | (234) | (419) | (212) | (252) | (280) |  |

a. For *Wolbachia* strains recorded by the *Wolbachia* PubMLST database, detailed information can be retrieved according to the isolate ID numbers indicated in Figure 1 and Figure S9.

b. The host species identified in this study were marked in bold, and their *Wolbachia* supergroups were determined from the MLST phylogeny.

c. PI: parthenogenesis induction; M: mutualism; P: parthenogenetic reproduction.

d. Sequences were represented by their their GenBank accession numbers and their allele numbers in *Wolbachia* PubMLST database in parentheses.

**Table S4.** Species analyzed in host phylogeny.

| Classification | Species | Accessions ^a^ | | |
| --- | --- | --- | --- | --- |
|  |  | mt*COI* | 18S rRNA | 28SrRNA |
| Class Collembola |  |  |  |  |
| Order Poduromorpha |  |  |  |  |
| Family Onychiuridae |  |  |  |  |
| Genus *Thalassaphorura* | *T. houtanensis* | **KT799635** | **KT799627** | **KT799632** |
| Family Tullbergiidae |  |  |  |  |
| Genus *Mesaphorura* | *Mes. yosii* | **KT799636** | **KT799628** | **KT799633** |
| Order Entomobryomorpha |  |  |  |  |
| Family Isotomidae |  |  |  |  |
| Genus *Folsomia* | *F. candida* (DK) | JN981072 | **KT799625** | **KT799630** |
| Genus *Folsomides* | *Fd. parvulus* | JN981069 | **KT799626** | **KT799631** |
| Order Neelipleona |  |  |  |  |
| Family Neelidae |  |  |  |  |
| Genus *Megalothorax* | *Meg. incertus* | **KT799634** | **KT799624** | **KT799629** |
|  |  |  |  |  |
| Class Protura |  |  |  |  |
| Order Acerentomata |  |  |  |  |
| Family Berberentulidae |  |  |  |  |
| Genus *Baculentulus* | *B. tianmushanensis* | Unpublished | AY037169 | AF416872 |
|  |  | data |  |  |
| Class (Order) Diplura |  |  |  |  |
| Family Campodeidae |  |  |  |  |
| Genus *Lepidocampa* | *L. weberi* | NC_022675 | AY037167 | AF416870 |
| Family Octostigmatidae |  |  |  |  |
| Genus *Octostigma* | *O. sinensis* | NC_022672 | AY145134 | AY596392 |
| a. Sequences obtained in this study were shown in bold. | | |  |  |

**Table S5**. Recombinants detected with RDP3 (Martin et al. 2010) and excluded from the phylogenetic analyses. ^a^

| Dataset | Gene | Recombination Event Number | Detection Methods | | Recombinant Sequence | Parental Sequences ^d^ | | | |
| --- | --- | --- | --- | --- | --- | --- | --- | --- | --- |
|  |  |  |  |  |  | Minor | | Major | |
| MLST ^b^ | *coxA* | 1 | Maxchi, SiScan | | 283_B | 73_B | | 320_B | |
|  |  | 2 | SiScan, 3Seq | | 92_B | 269_B | | 24_B | |
|  |  |  |  | | 73_B | 267_B | | 19_B | |
|  | *fbpA* | 1 | Maxchi, Chimaera, SiScan, 3Seq | | 296_B | 27_B | | 352_A | |
|  |  |  |  | | 22_B | 19_B | | 1_A | |
|  |  |  |  | | 24_B | 21_B | | 2_A | |
|  |  |  |  | | 28_B | 25_B | | 3_A | |
|  |  |  |  | | 320_B | 26_B | | 4_A | |
|  |  | 2 | Bootscan, Maxchi, 3Seq | | 342_B | 468_B | | 68_A | |
|  |  |  |  | | 23_B | 25_B | | 1_A | |
|  |  |  |  | | 34_B | 29_B | | 3_A | |
|  |  |  |  | | 39_B | 31_B | | 4_A | |
|  |  |  |  | | 73_B | 33_B | | 5_A | |
|  |  |  |  | | 99_B | 40_B | | 6_A | |
|  |  |  |  | | 312_B | 70_B | | 7_A | |
|  |  |  |  | | 314_B | 132_B | | 8_A | |
|  |  |  |  | | 316_B | 194_B | | 9_A | |
|  |  |  |  | | 319_B | 195_B | | 10_A | |
|  |  |  |  | | 423_B | 208_B | | 11_A | |
|  |  |  |  | | 471_B | 212_B | | 12_A | |
|  |  |  |  | |  |  | |  | |
| **Table S5.** Continued. | | | | | | | | | |
| Dataset | Gene | Recombination Event Number | Detection Methods | | Recombinant Sequence | Parental Sequences ^d^ | | |  |
|  |  |  |  |  |  | Minor | Major | |  |
| MLST ^b^ | *ftsZ* | 1 | SiScan, 3Seq | | 105_A | 55_A | 24_B | | |
|  |  |  |  | | 3_A | 1_A | 24_B | | |
|  |  |  |  | | 109_A | 12_A | 24_B | | |
|  |  |  |  | | 134_A | 15_A | 24_B | | |
|  |  |  |  | | 558_A | 18_A | 24_B | | |
|  |  |  |  | |  |  |  | | |
| WSP ^c^ | *wsp* | 1 | GENECONV, Maxchi, Chimaera, SiSscan, 3Seq | | 103_A | 168_A | 555_A | | |
|  |  |  |  | | 142_A | 3_A | 1_A | | |
|  |  | 2 | GENECONV, Maxchi, Chimaera, SiSscan, 3Seq | | 110_A | 138_A | 352_A | | |
|  |  |  |  | | 78_A | 11_A | 3_A | | |
|  |  | 3 | GENECONV, Maxchi, Chimaera, SiSscan, 3Seq | | 13_A | 104_A | 137_A | | |
|  |  |  |  | | 38_A | 4_A | 251_other | | |
|  |  |  |  | | 111_A | 11_A | 251_other | | |
|  |  |  |  | | 116_A | 18_A | 251_other | | |
|  |  |  |  | | 135_A | 96_A | 251_other | | |
|  |  |  |  | | 141_A | 113_A | 251_other | | |
|  |  |  |  | | 399_A | 120_A | 251_other | | |
|  |  |  |  | | 401_A | 129_A | 251_other | | |
|  |  |  |  | | 613_A | 133_A | 251_other | | |
|  |  | 4 | Maxchi, Chimaera, SiSscan, 3Seq | | 351_A | 15_A | 139_A | | |
|  |  | 5 | Maxchi, Chimaera, SiSscan, 3Seq | | 137_A | 251_other | 420_B | | |
|  |  |  |  | |  |  |  | | |
| **Table S5.** Continued. | | | | | | | | | |
| Dataset | Gene | Recombination Event Number | Detection Methods | Recombinant Sequence | | Parental Sequences ^d^ | | |  |
|  |  |  |  |  |  | Minor | Major | |  |
| WSP ^c^ | *wsp* | 6 | Maxchi, Chimaera, SiSscan, 3Seq | 17_A | | 11_A | 113_A | | |
|  |  |  |  | 112_A | | 250_A | 14_A | | |
|  |  |  |  | 294_A | | 250_A | 14_A | | |
|  |  |  |  | 325_A | | 250_A | 14_A | | |
|  |  | 7 | Maxchi, Chimaera | 118_B | | 352_A | 251_other | | |
|  |  |  |  | 309_B | | 352_A | 251_other | | |
|  |  | 8 | Maxchi, SiSscan | 133_A | | 250_A | 164_F | | |
|  |  |  |  | 98_A | | 9_A | 42_F | | |
|  |  |  |  | 145_A | | 61_A | 44_F | | |

a. Test for recombination signals was conducted for the MLST dataset first, and then for the *wsp* genes of strains without recombinant MLST loci. Recombination events detected by more than two methods and recombinants with both parental sequences determined were accepted. *Wolbachia* strains are indicated with their isolate IDs and supergroup annotations.

b. No recombinant was found for *gatB* and *hcpA* genes.

c. For the presumed *wsp* recombinants, the actual breakpoint position, either the beginning or the end breakpoint is undetermined, suggesting that it was most likely overprinted by a subsequent recombination event.

d. For each recombinant, only one pair of possible parents is shown here. Minor parent: parent contributing the smaller fraction of sequence; major parent: parent contributing the larger fraction of sequence.

**Table S6.** Best-fit partitioning schemes and nucleotide substitution models for phylogenetic analyses ^a^.

| Dataset |  | Partitioning scheme | | Best model for |
| --- | --- | --- | --- | --- |
|  |  | Subset | Corresponding sites | Bayesian inference |
| 16S rRNA+*ftsZ* | combined dataset | 1 | 16S rRNA; *ftsZ*, 2nd codon positition | K80+I+G |
|  |  | 2 | *ftsZ*, 1st codon positition | GTR+G |
|  |  | 3 | *ftsZ*, 3rd codon positition | GTR+I+G |
|  | 16S rRNA |  | (no partitioning) | HKY+I+G |
|  | *ftsZ* | 1 | *ftsZ*, 1st codon positition | GTR+G |
|  |  | 2 | ftsZ, 2nd codon positition | K80+I+G |
|  |  | 3 | ftsZ, 3rd codon positition | GTR+I+G |
| MLST | combined dataset | 1 | five MLST loci, 1st codon positition | GTR+I+G |
|  |  | 2 | five MLST loci, 2nd codon positition | HKY+I+G |
|  |  | 3 | five MLST loci, 3rd codon positition | GTR+I+G |
|  | *coxA* | 1 | *coxA*, 1st codon positition | K80+G |
|  |  | 2 | *coxA*, 2nd codon positition | HKY+G |
|  |  | 3 | *coxA*, 3rd codon positition | GTR+I+G |
|  | *fbpA* | 1 | *fbpA*, 1st codon positition | GTR+G |
|  |  | 2 | *fbpA*, 2nd codon positition | HKY+I+G |
|  |  | 3 | *fbpA*, 3rd codon positition | GTR+G |
|  | *ftsZ* | 1 | *ftsZ*, 1st codon positition | GTR+G |
|  |  | 2 | *ftsZ*, 2nd codon positition | K80+I+G |
|  |  | 3 | *ftsZ*, 3rd codon positition | HKY+I+G |
|  | *gatB* | 1 | *gatB*, 1st and 2nd codon positions | HKY+G |
|  |  | 2 | *gatB*, 3rd codon positition | GTR+G |
|  | *hcpA* | 1 | *hcpA*, 1st and 2nd codon positions | K80+G |
|  |  | 2 | *hcpA*, 3rd codon positition | GTR+G |
| *wsp* |  | 1 | *wsp*, 1st codon positition | GTR+I+G |
|  |  | 2 | *wsp*, 2nd codon positition | GTR+I+G |
|  |  | 3 | *wsp*, 3rd codon positition | GTR+G |
| Hosts' genes |  | 1 | mt*COI* | GTR+G |
|  |  | 2 | 18S rRNA, 28S rRNA | GTR+G |
| a. Maximum likelihood analyses were all conducted under the GTRGAMMA model. | | | |  |


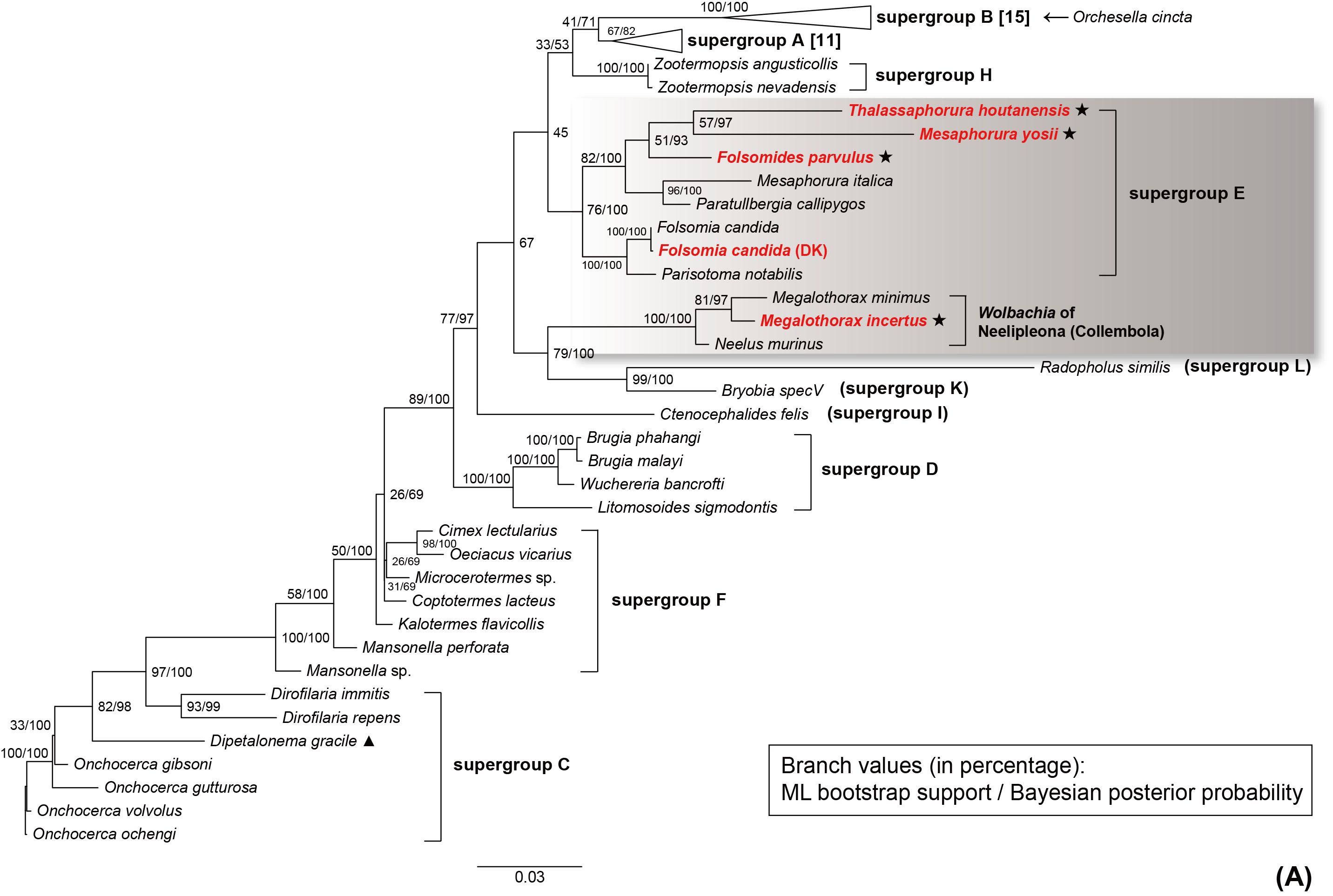


**Figure S7. Unrooted phylogenetic trees obtained using maximum likelihood estimation based on *Wolbachia* 16S rRNA and *ftsZ* gene sequences.**

*Wolbachia* strains (Table S2) are represented with the name of their host species, and strains recovered in this study are indicated in red and bold. Strains infecting parthenogenetic collembolan species are highlighted; the *Wolbachia* from collembolans collected in China are indicated by solid pentagrams.

**A**. ML tree based on the concatenated dataset covering 60 taxa, including all eleven *Wolbachia* strains of parthenogenetic collembolans. The Bayesian tree (not shown) exhibited a similar topology, with slight differences within clades A and B. The support values at the nodes indicate ML bootstraps (left) and Bayesian posterior probabilities (right). Supergroups A and B are collapsed, with the number of sequences labeled in brackets. The position of the B-type *Wolbachia* endosymbiont of the bisexual springtail *Ochesella cincta* is noted. The strain marked with a triangle was previously reported to be the sole representative of supergoup J.

**B**. ML tree based on the concatenated dataset covering 57 taxa, with supergroups I, K, L excluded. The support values at the nodes indicate ML bootstraps.

**C.** ML tree based on a concatednated dataset of 54 taxa. For *Wolbachia* infecting parthenogenetic collembolans, only the five strains recoved in this study were included.

**D**. ML tree based only on the 16S rRNA gene covering 60 taxa. The co-infected A-type and B-type *Wolbachia* strains of the *Nasonia* wasps *N. longicornis* and *N. vitripennis*, respectively, are distinguished by their GenBank accession numbers in single gene trees.

**E**. ML tree based only on the *ftsZ* gene covering 60 taxa.


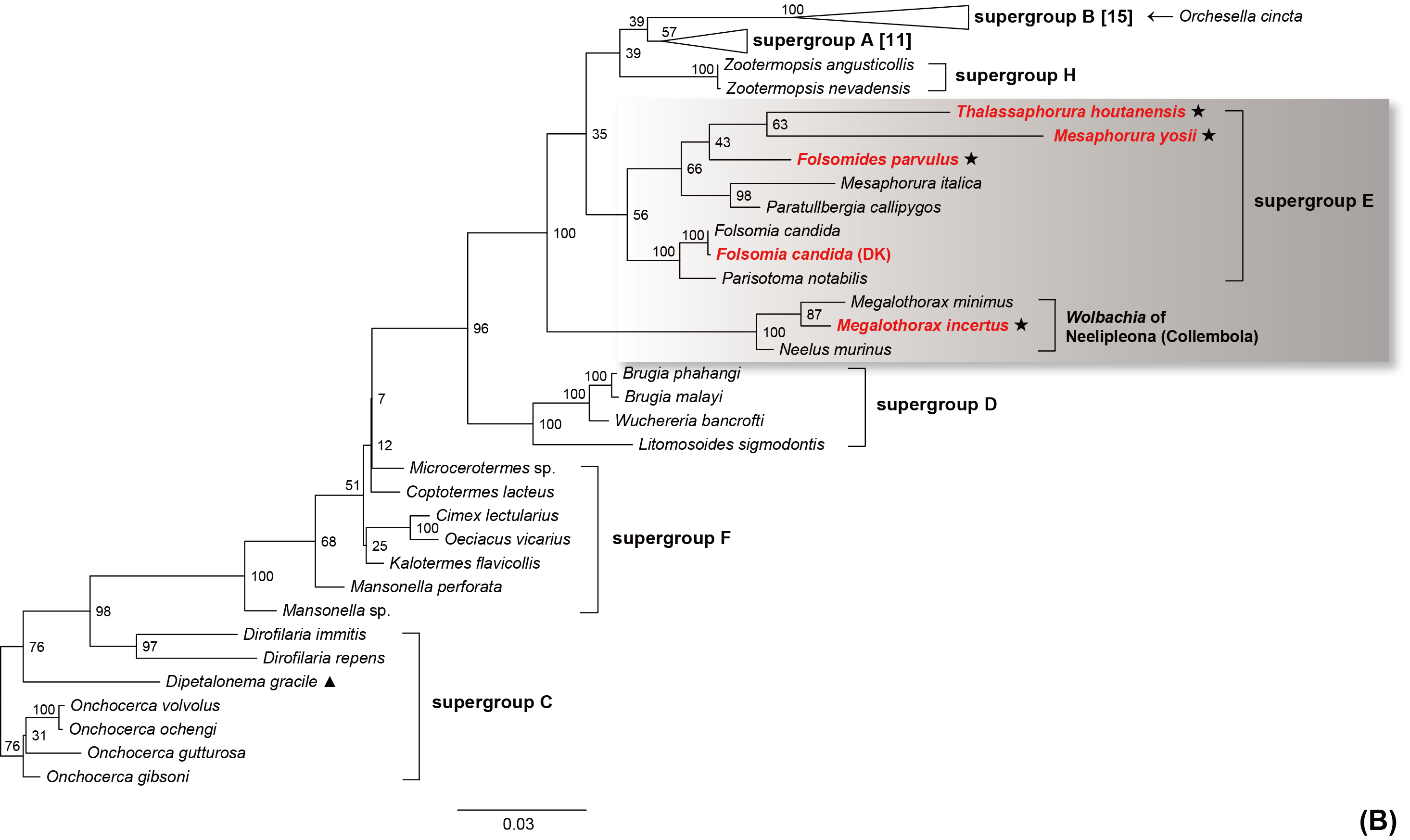


**Figure S7.** Continued.


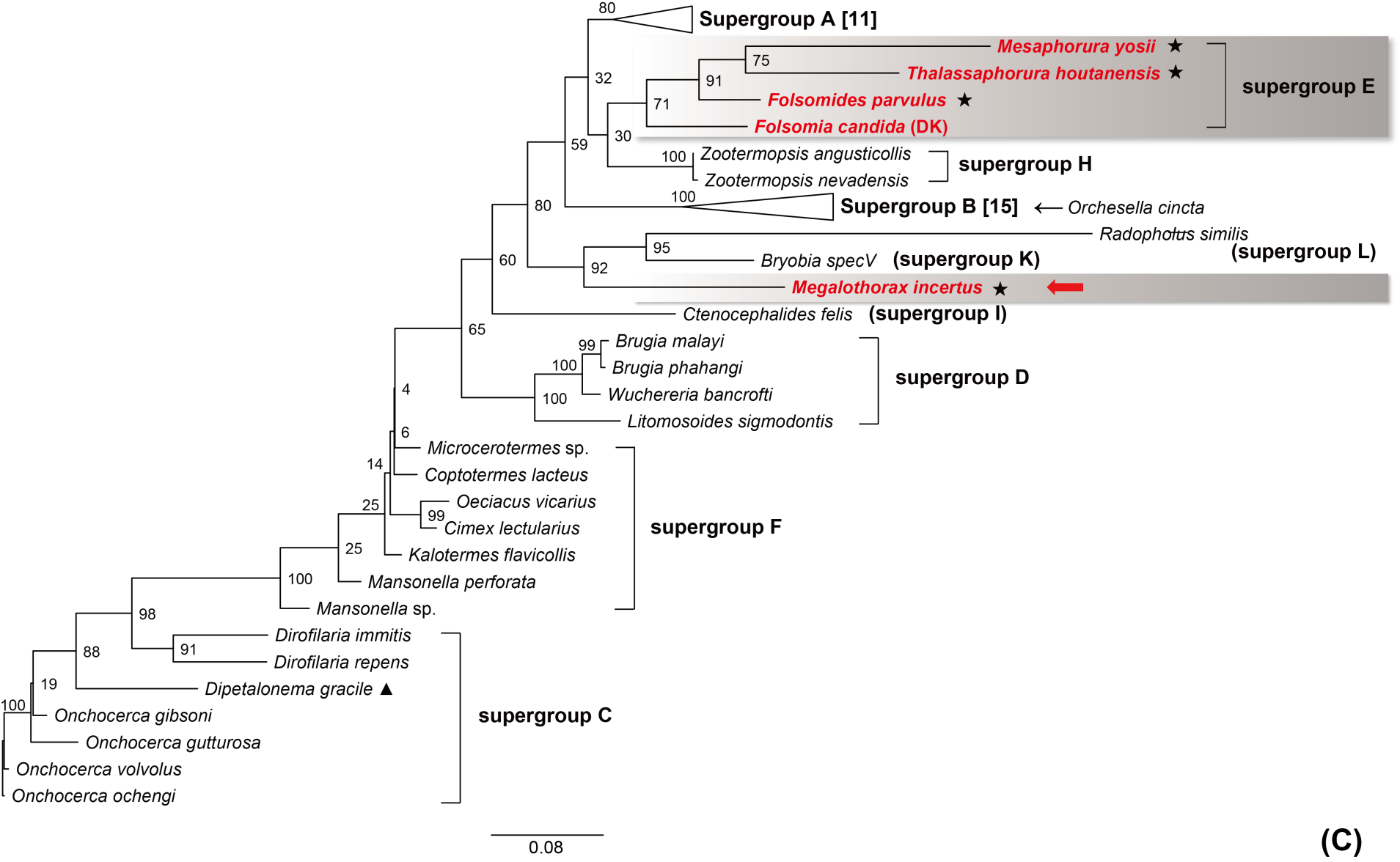


**Figure S7.** Continued.


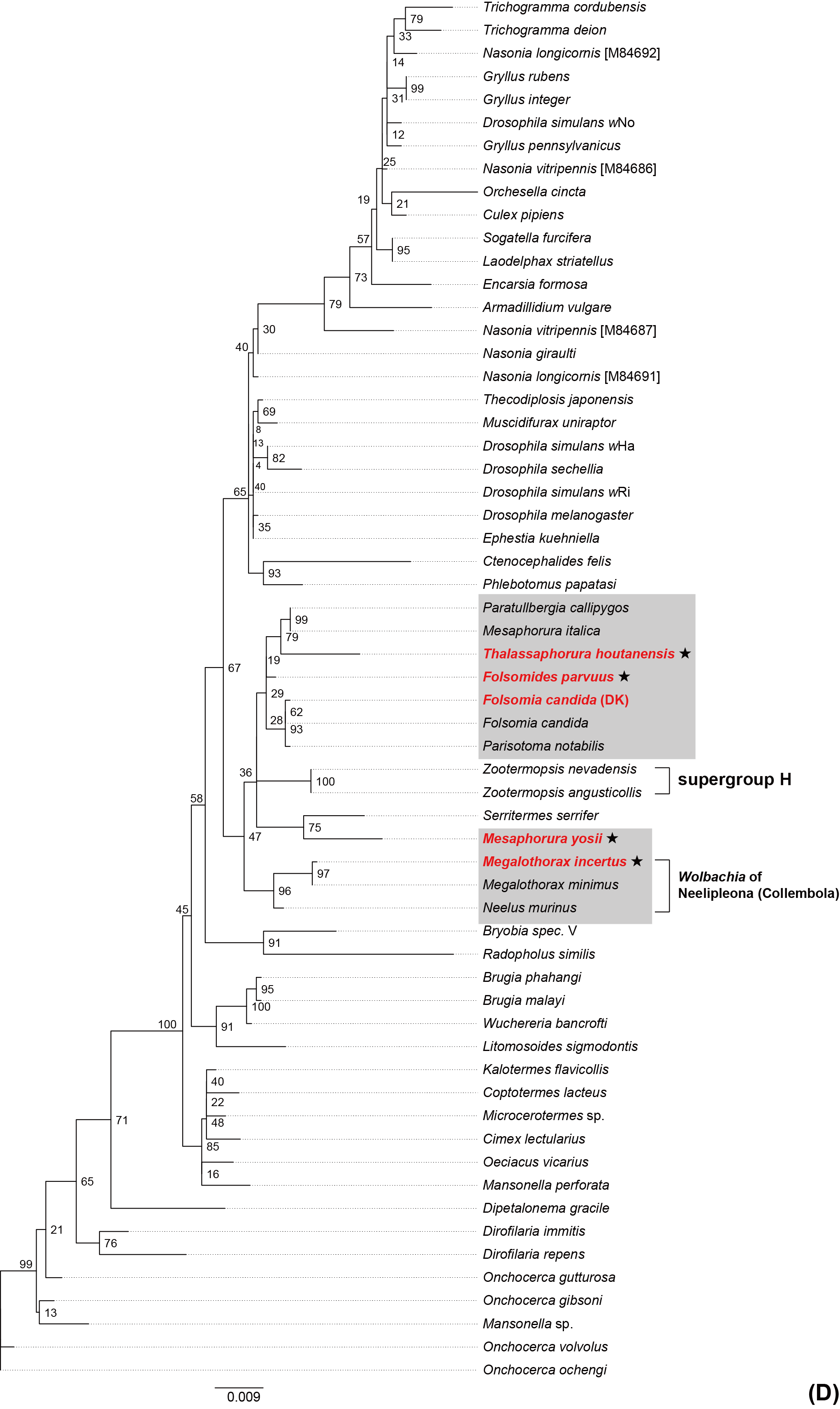


**Figure S7.** Continued.


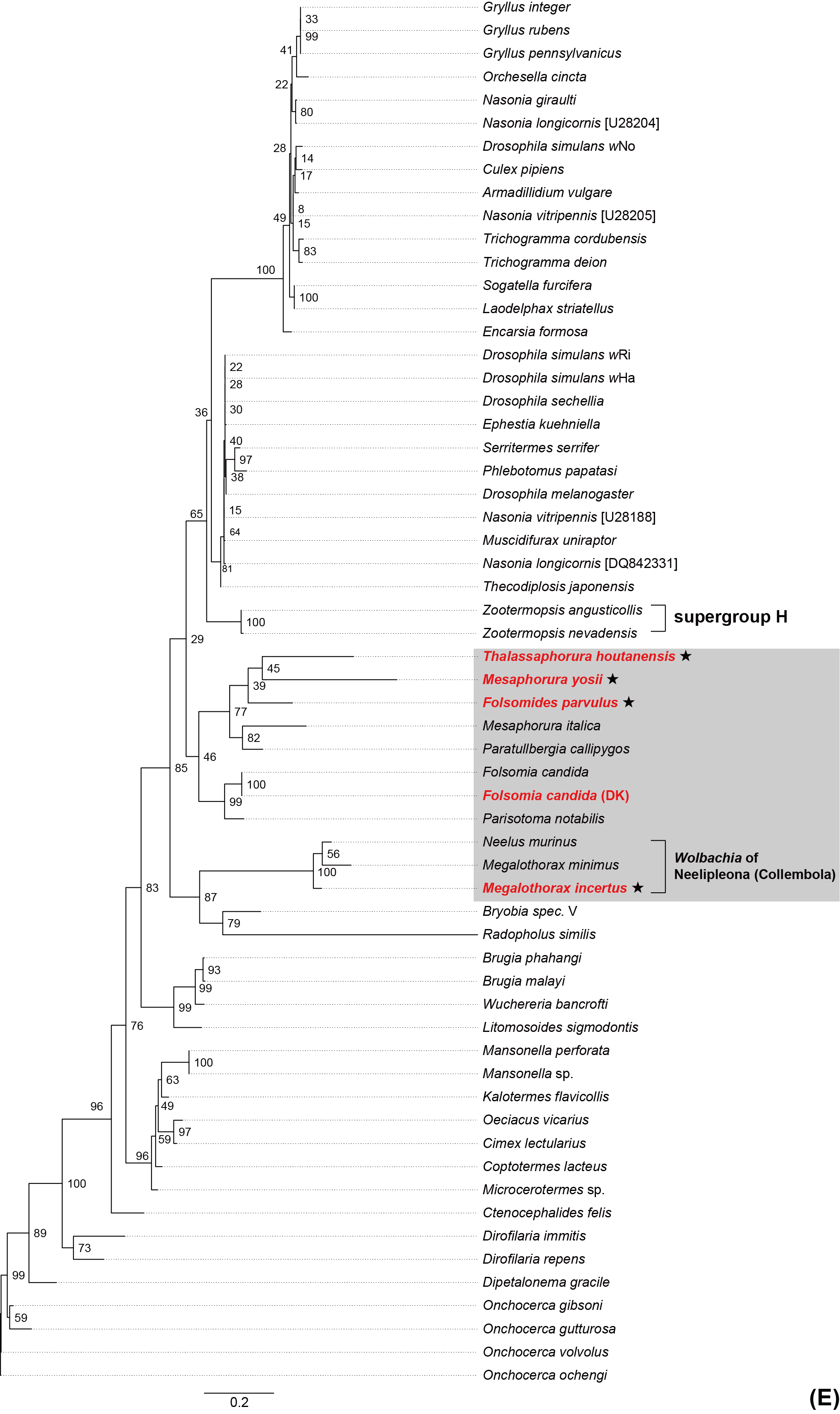


**Figure S7.** Continued.


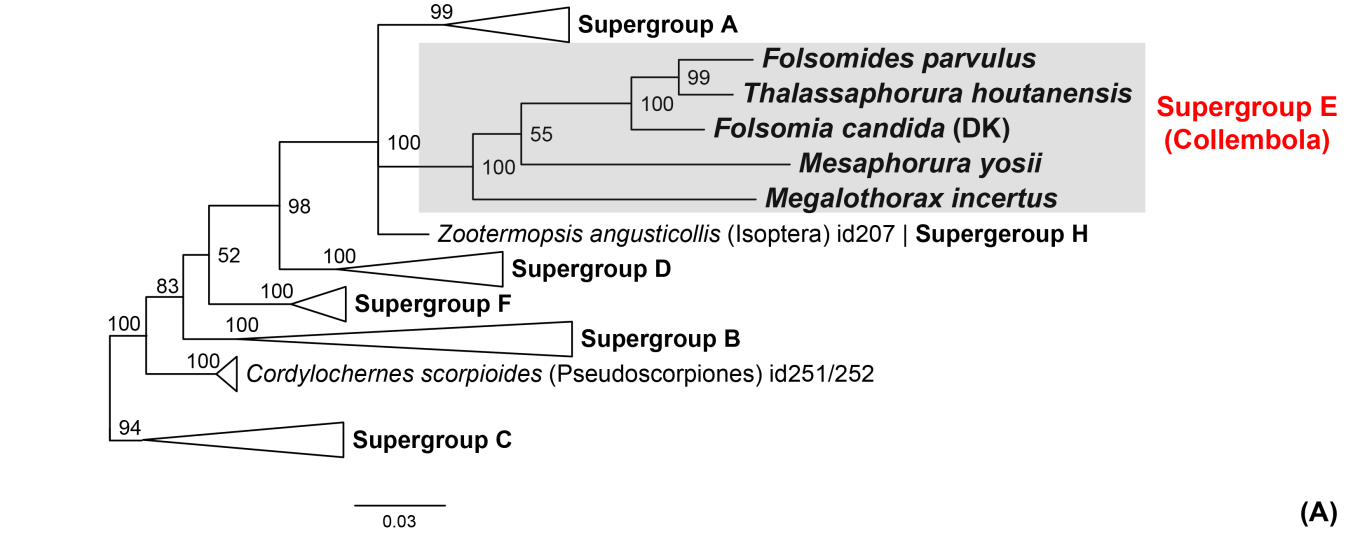


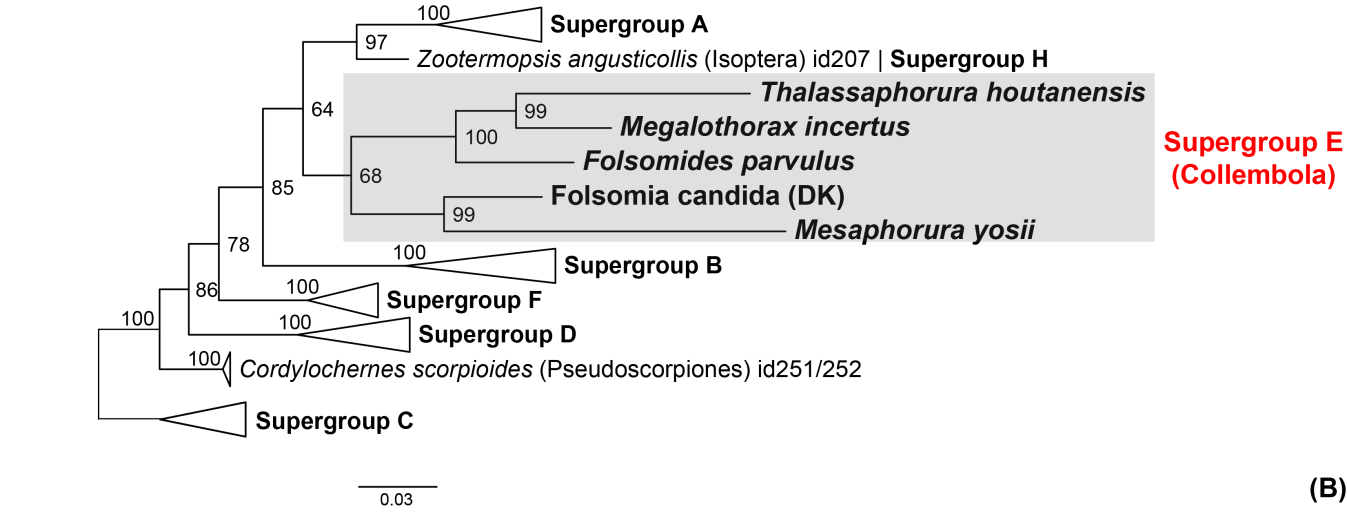


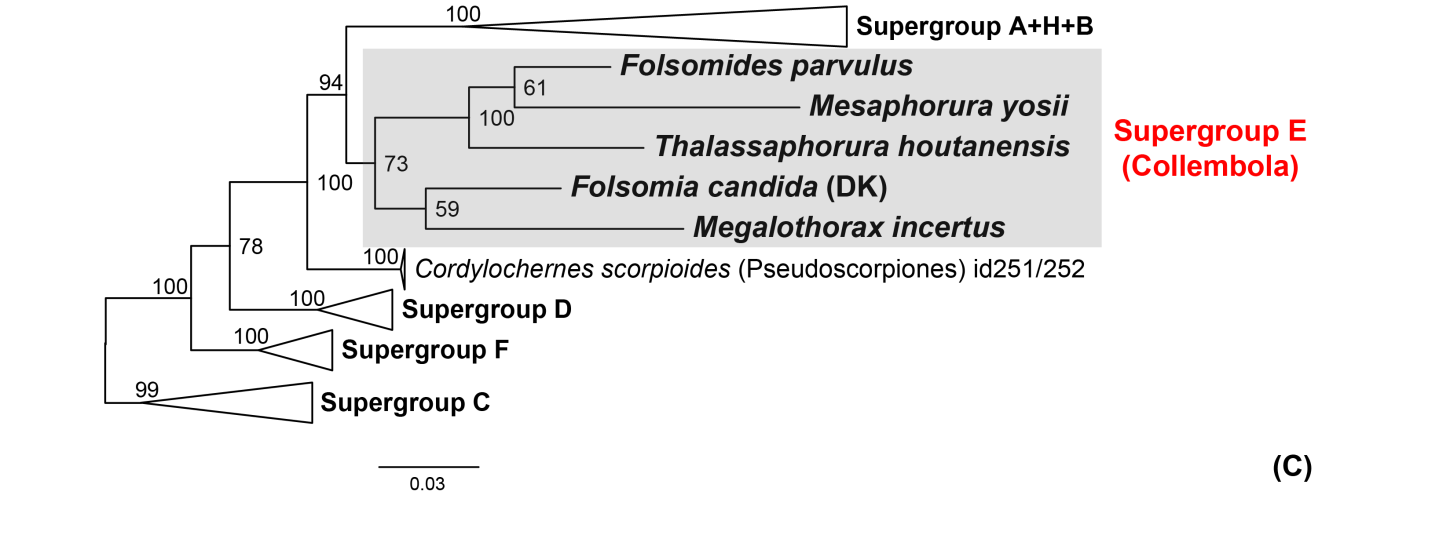


**Figure S8. Unrooted Bayesian trees inferred from five single genes of the *Wolbachia* MLST system.**

(A) *coxA*; (B) *fbpA*; (C) *ftsZ*; (D) *gatB*; (E) *hcpA*. Support values at nodes indicate Bayesian posterior probabilities in percentages. Most clades are collapsed to simplify the display of trees, except for supergroup E.


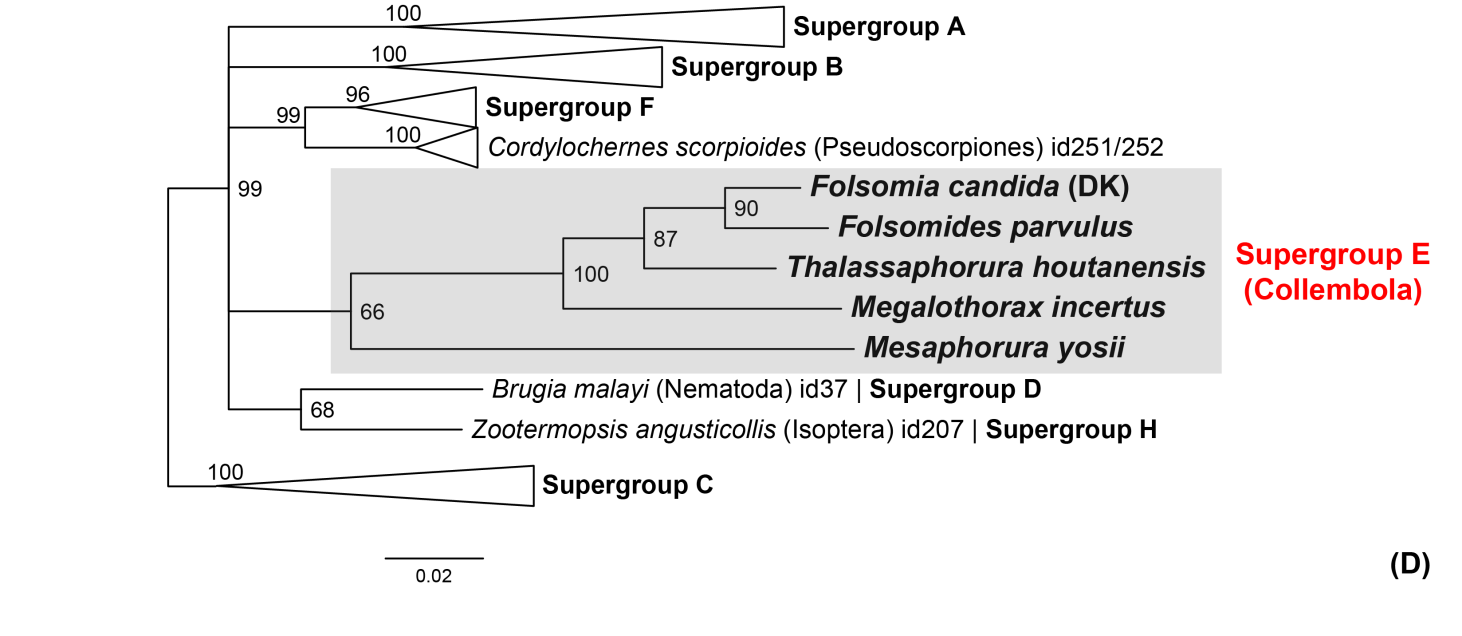


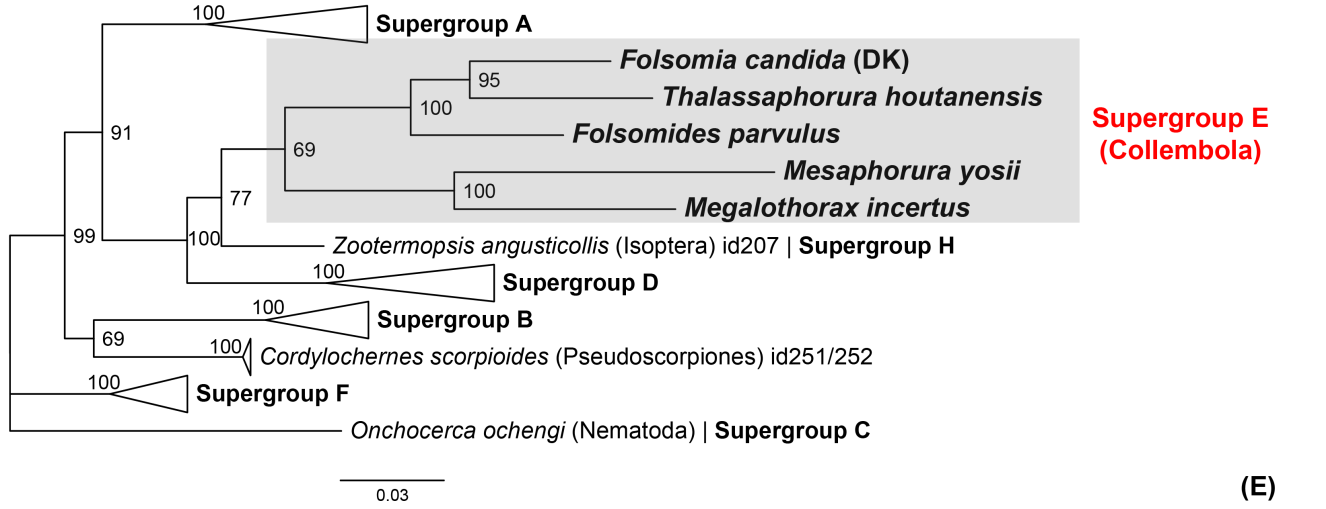


**Figure S8.** Continued.


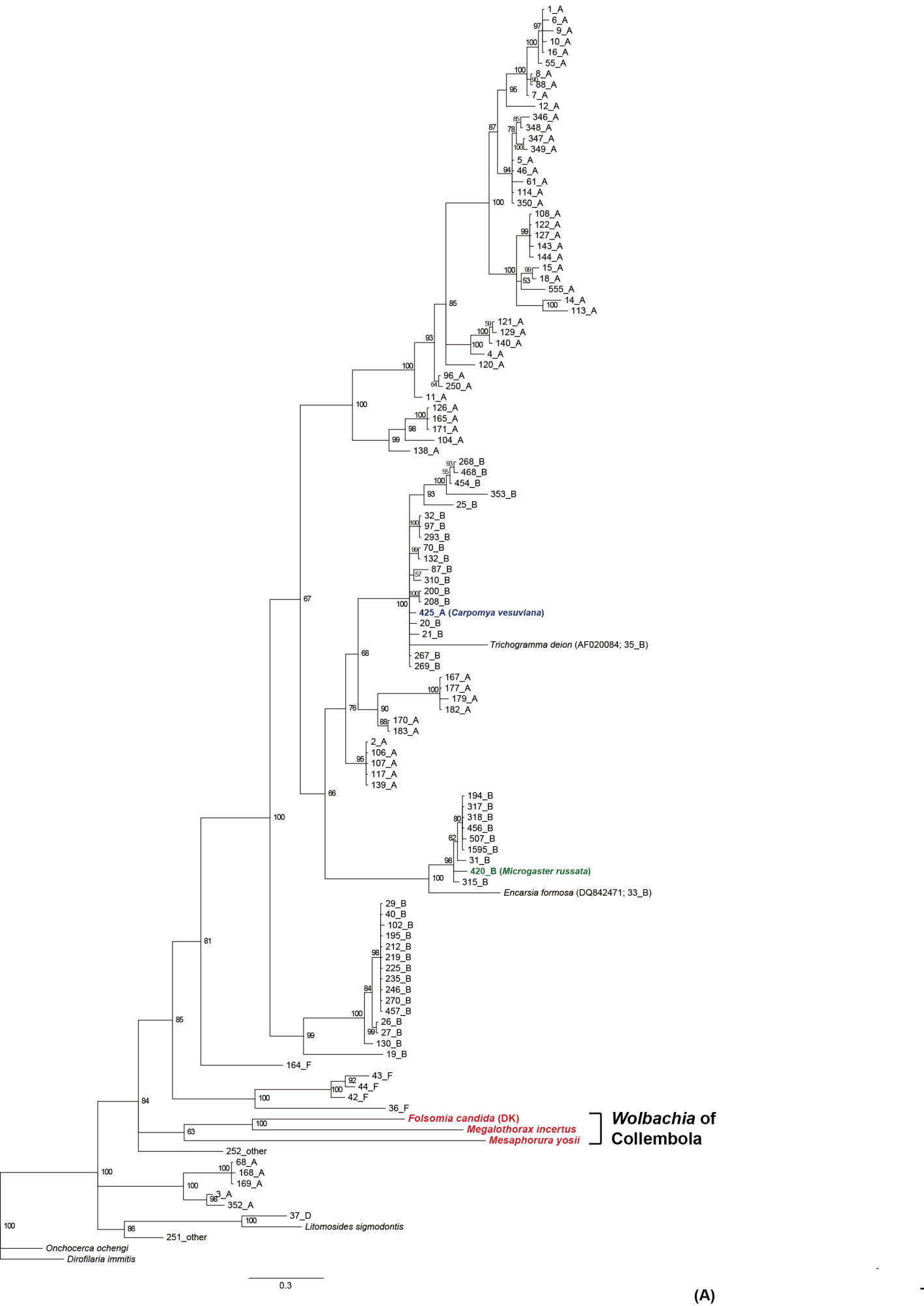


**Figure S9. Phylogenetic trees based on *wsp* gene sequences.**

Bayesian (A) and ML (B) trees were reconstructed for the 117-taxon *wsp* nucleotide alignment that passed recombination detection. Sequences downloaded from PubMLST are represented by the ID numbers of their strains, followed by the supergroup annotation. Sequences retrieved from GenBank (including sequences for isolate 33 and 35) are indicated by their host species names. Data obtained in this study are presented with the host species names and are highlighted in red. A monophyletic clade was recovered for the three collembolan *Wolbachia* strains using both inference methods. As a note, a mismatch between the MLST and *wsp* data was found for isolate 420 and 425 in our study: MLST typing assigned both isolates to supergroup A, while *wsp* typing assigned both of them to supergroup B. Correpsonding *wsp* sequences for isolate 420 and 425 are indicated separately in green and blue.


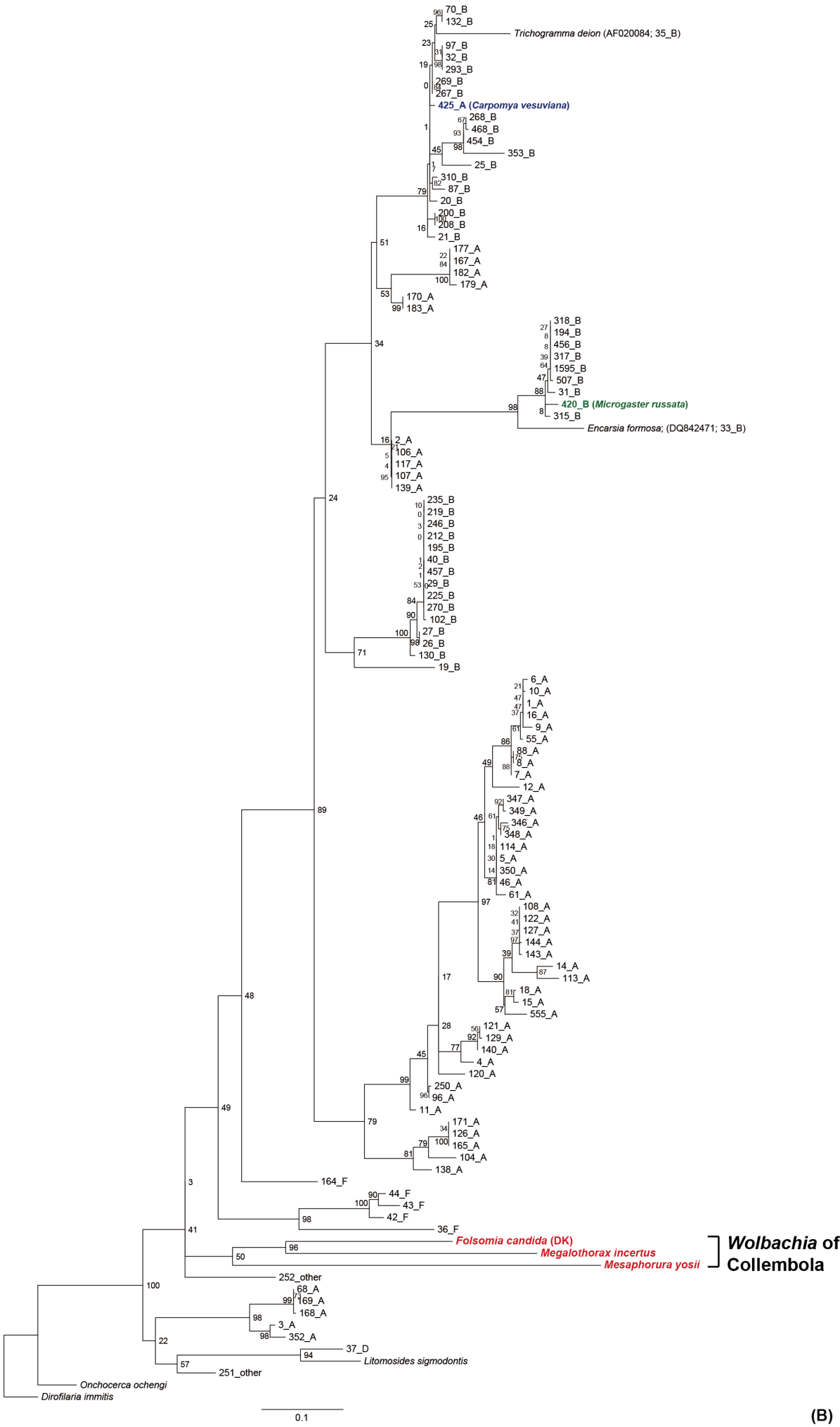


**Figure S9.** Continued.

**Supplementray file 10.** References cited in Table S1.

Baldo, L., S. Bordenstein, J. J. Wernegreen, and J. H. Werren. 2006. Widespread recombination throughout *Wolbachia* genomes. Mol. Biol. Evol. 23:437-449.

Casiraghi, M., S. R. Bordenstein, L. Baldo, N. Lo, T. Beninati, J. J. Wernegreen, J. H. Werren, and C. Bandi. 2005. Phylogeny of *Wolbachia pipientis* based on *gltA*, *groEL* and *ftsZ* gene sequences: clustering of arthropod and nematode symbionts in the F supergroup, and evidence for further diversity in the *Wolbachia* tree. Microbiology 151:4015-4022.

Czarnetzki, A. B. and C. C. Tebbe. 2004. Detection and phylogenetic analysis of *Wolbachia* in Collembola. Environ. Microbiol. 6:35-44.

Folmer, O., M. Black, W. Hoeh, R. Lutz, and R. Vrijenhoek. 1994. DNA primers for amplification of mitochondrial cytochrome c oxidase subunit I from diverse metazoan invertebrates. Mol. Mar. Biol. Biotechnol. 3:294-299.

Gavotte, L., H. Henri, R. Stouthamer, D. Charif, S. Charlat, M. Bouletreau, and F. Vavre. 2007. A Survey of the bacteriophage WO in the endosymbiotic bacteria *Wolbachia*. Mol. Biol. Evol. 24:427-435.

Jeyaprakash, A. and M. A. Hoy. 2000. Long PCR improves *Wolbachia* DNA amplification: *wsp* sequences found in 76% of sixty-three arthropod species. Insect Mol. Biol. 9:393-405.

Luan, Y. X., J. M. Mallatt, R. D. Xie, Y. M. Yang, and W. Y. Yin. 2005. The phylogenetic positions of three Basal-hexapod groups (Protura, Diplura, and Collembola) based on ribosomal RNA gene sequences. Mol. Biol. Evol. 22:1579-1592.

O'Neill, S. L., R. Giordano, A. M. Colbert, T. L. Karr, and H. M. Robertson. 1992. 16S rRNA phylogenetic analysis of the bacterial endosymbionts associated with cytoplasmic incompatibility in insects. Proc. Natl. Acad. Sci. U. S. A. 89:2699-2702.

Tanganelli, V., P. P. Fanciulli, F. Nardi, and F. Frati. 2014. Molecular phylogenetic analysis of a novel strain from Neelipleona enriches *Wolbachia* diversity in soil biota. Pedobiologia 57:15-20.

Werren, J. H., W. Zhang, and L. R. Guo. 1995. Evolution and phylogeny of *Wolbachia*: reproductive parasites of arthropods. Proc. R. Soc. Lond., B, Biol. Sci. 261:55-63.

Zhou, W., F. Rousset, and S. O'Neil. 1998. Phylogeny and PCR-based classification of *Wolbachia* strains using *wsp* gene sequences. Proc. R. Soc. Lond., B, Biol. Sci. 265:509-515.
